# Control of ϕC31 integrase-mediated site-specific recombination by protein *trans*-splicing

**DOI:** 10.1093/nar/gkz936

**Published:** 2019-10-31

**Authors:** Femi J Olorunniji, Makeba Lawson-Williams, Arlene L McPherson, Jane E Paget, W Marshall Stark, Susan J Rosser

**Affiliations:** 1 School of Pharmacy and Biomolecular Sciences, Faculty of Science, Liverpool John Moores University, James Parsons Building, Byrom Street, Liverpool L3 3AF, UK; 2 Institute of Molecular, Cell and Systems Biology, University of Glasgow, Bower Building, Glasgow G12 8QQ, UK; 3 UK Centre for Mammalian Synthetic Biology at the Institute of Quantitative Biology, Biochemistry, and Biotechnology, SynthSys, School of Biological Sciences, University of Edinburgh, Edinburgh, EH9 3JD, UK; 4 Institute for Bioengineering, University of Edinburgh, Faraday Building, The King's Buildings, Edinburgh, 2 EH9 3DW, UK

## Abstract

Serine integrases are emerging as core tools in synthetic biology and have applications in biotechnology and genome engineering. We have designed a split-intein serine integrase-based system with potential for regulation of site-specific recombination events at the protein level *in vivo*. The ϕC31 integrase was split into two extein domains, and intein sequences (*Npu* DnaE**^N^** and *Ssp* DnaE**^C^**) were attached to the two termini to be fused. Expression of these two components followed by post-translational protein *trans*-splicing in *Escherichia coli* generated a fully functional ϕC31 integrase. We showed that protein splicing is necessary for recombination activity; deletion of intein domains or mutation of key intein residues inactivated recombination. We used an invertible promoter reporter system to demonstrate a potential application of the split intein-regulated site-specific recombination system in building reversible genetic switches. We used the same split inteins to control the reconstitution of a split Integrase-Recombination Directionality Factor fusion (Integrase-RDF) that efficiently catalysed the reverse *attR* x *attL* recombination. This demonstrates the potential for split-intein regulation of the forward and reverse reactions using the integrase and the integrase-RDF fusion, respectively. The split-intein integrase is a potentially versatile, regulatable component for building synthetic genetic circuits and devices.

## INTRODUCTION

It has recently become possible to create computational and memory systems in cells (1–3) allowing us to foresee many new ways to enhance the applications of living organisms (4,5). Engineered cells could act as powerful biosensors with applications in health, environmental and industrial processes. As well as sensing components, it is necessary to process the received information and express it as outputs in the form of specific biological responses (3,6). However, the genetic switches and logic gates that have been constructed to date are based on a limited repertoire of biological component types (7), and there is a need for new systems that can be used to implement more elaborate and robust devices.

**Figure 1.**
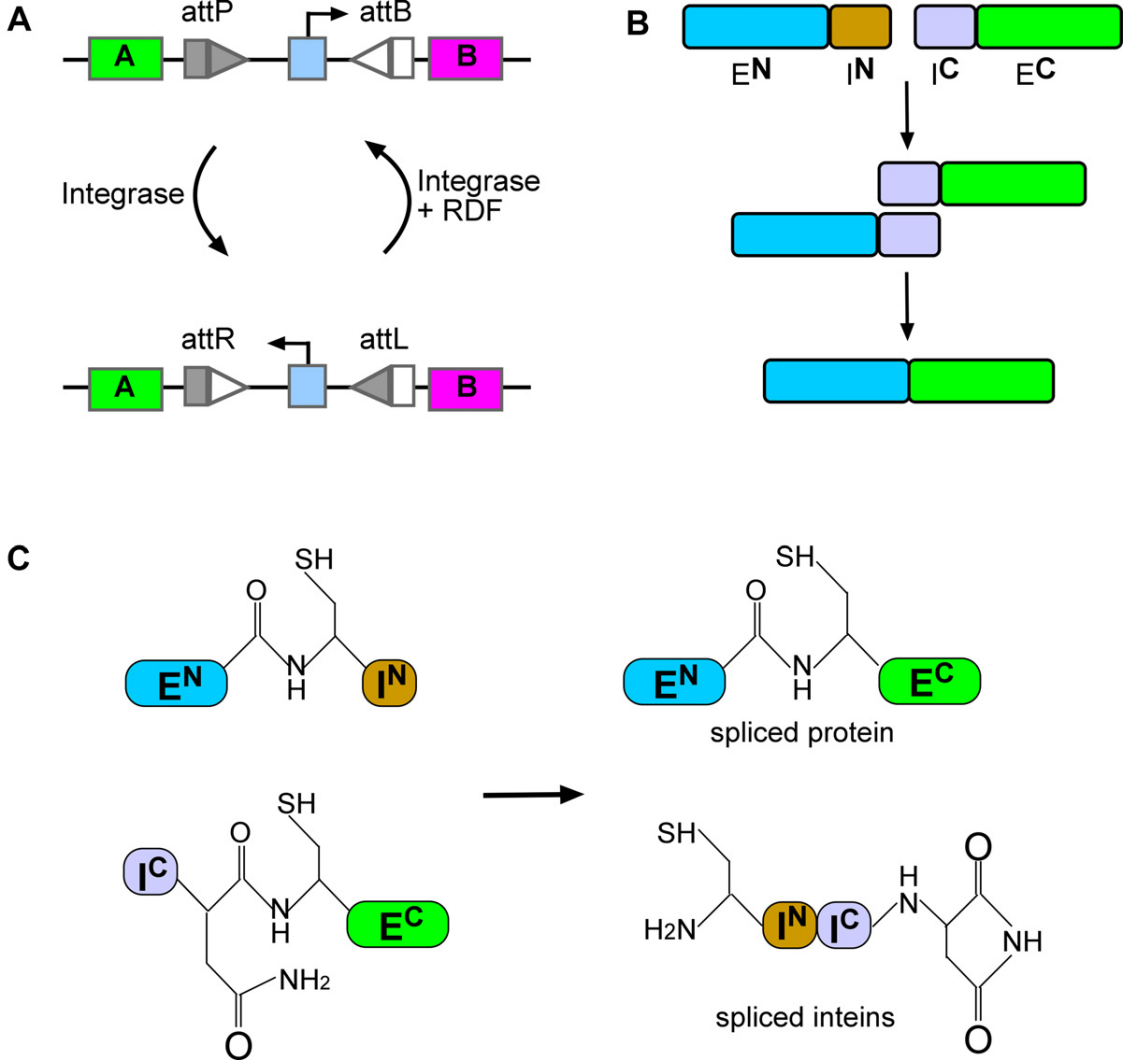
Building blocks for split intein-mediated *trans*-splicing of ϕC31 integrase. (**A**) Serine integrase catalysed inversion of the orientation of a promoter sequence functions as a toggle switch for the expression of two genes. Integrase catalysed recombination between *attP* and *attB* sites (arranged in inverted orientation) results in switching the direction of the promoter, and hence changes the gene expression status of the module. Recombination of *attR* and *attL* sites by integrase and the RDF, restores the module to its original state. (**B**) Split-intein catalysed protein *trans*-splicing. Non-covalent association of the N-terminal intein (**I^N^**) and the C-terminal intein (**I^C^**) is followed by a *trans*-splicing reaction that covalently joins the N-terminal extein (**E^N^**) and C-terminal extein (**E^C^**). (**C**) Intein-mediated protein *trans*-splicing involves specific chemical steps requiring a catalytic nucleophile. In this example, cysteine residues are required to mediate the reactions.

DNA site-specific recombination has been much exploited for rapid DNA assembly, and to build genetic switches and memory devices (4,8–10). In a typical module, two recombination sites flank a promoter sequence. Expression of the recombinase promotes inversion of the orientation of the promoter sequence, thus switching between expression of two genes which are divergently transcribed from the module (Figure 1A). One group of site-specific recombinases known as the serine integrases is especially suited for the construction of switching devices, particularly because these enzymes promote very efficient and highly directional recombination (11). For such modules to be useful, fully integrated components of the cell, activity of the recombinase must be tightly regulated, so that switching occurs only when other cellular conditions are fulfilled. Here we demonstrate a powerful new approach to regulation of serine integrase activity, in which the enzyme itself is assembled by intein-mediated fusion of two precursor components.

Inteins are naturally occurring autocatalytic systems that catalyse protein splicing reactions to generate active proteins from precursor polypeptides (12). Synthetic ‘split inteins’ have been developed to carry out protein splicing *in trans*, covalently joining two proteins together for a wide range of biotechnological applications (13,14) (Figure 1B). For example, Schaerli *et al.* (15) split T7 polymerase into two parts and fused each part to split-intein sequences so that the two parts were covalently joined together by *trans*-splicing. Expression of each of the two individual parts was placed under the control of an inducible promoter to allow conditional expression of polymerase activity.

A key requirement for the construction of split-intein systems is the need to introduce an intein nucleophilic residue (typically Cys, Ser or Thr; Figure 1C) by mutation of the sequence of the target protein adjacent to the junction between the two ‘exteins’. Such changes can potentially lead to reduction or loss of enzyme activity. However, inteins have been engineered that tolerate variations of these flanking residues, thereby minimizing the number of changes that need to be made in the target protein (16,17).

Conditional site-specific recombinase activation by assembly of split protein fragments has been achieved; for example, for the popularly used tyrosine recombinase Cre (18–22). Wang *et al.* (22) used a Cre split-intein system to reconstitute functional recombinase in transgenic mice. However, there are no reports to date of analogous systems using split serine integrases. The transposase TnpX (distantly related to the serine integrases) was split into two parts and some DNA-binding activity was reconstituted when both parts were present, but no recombination (transposition) activity was observed (23).

With the increasing importance of serine integrases as tools in synthetic biology, methods for signal-induced post-translational regulation of integrase activity are becoming very desirable. Here, we report post-translational activation of site-specific recombination by reconstitution of functional ϕC31 integrase using split intein-catalysed reactions.

## MATERIALS AND METHODS

### Plasmids and DNA

**Figure 2.**
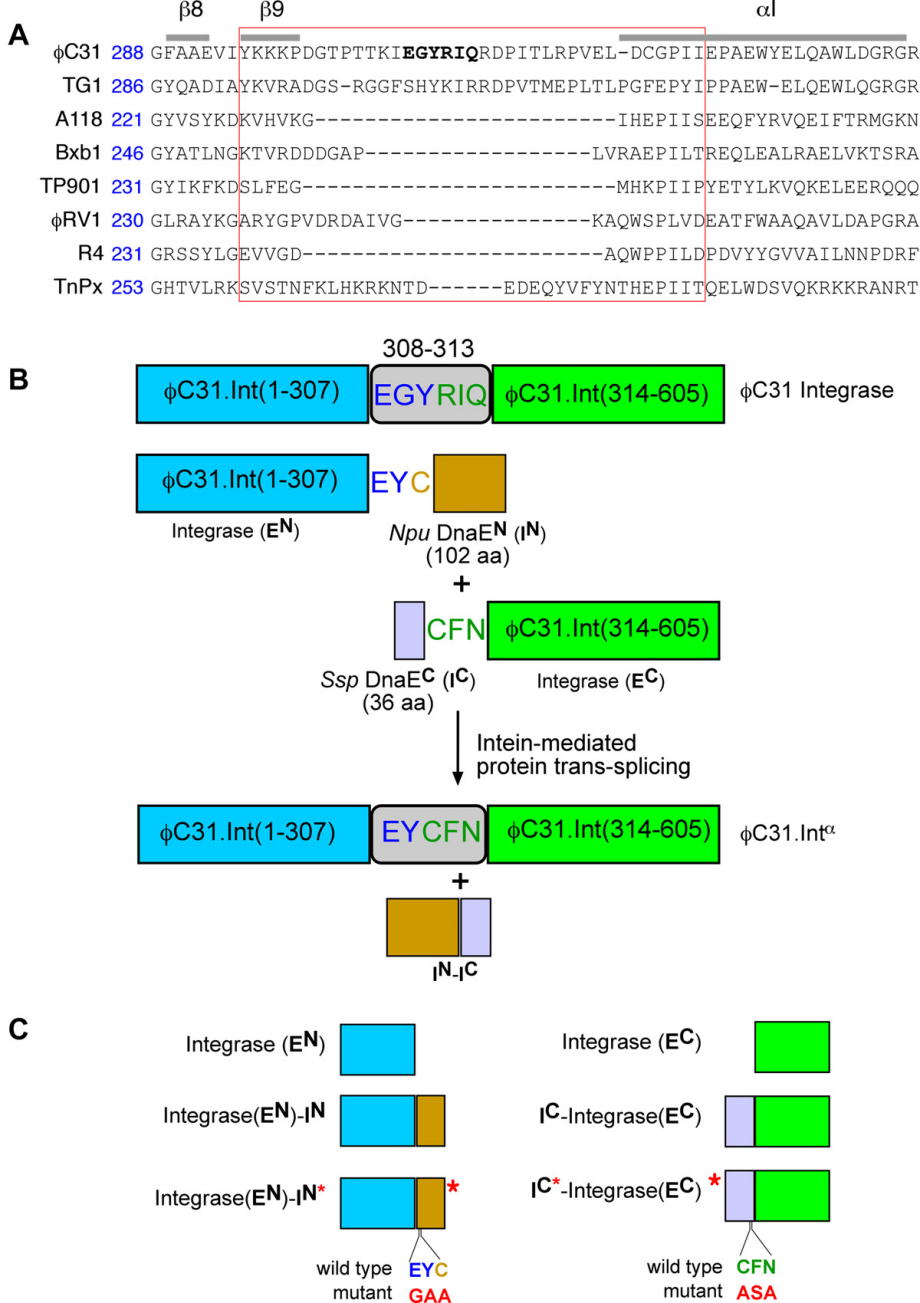
Design of split ϕC31 integrase. (**A**) Sequence alignment of ϕC31 integrase with related members of the ‘large serine recombinase’ protein family based on published structures and similar alignments^27^. Amino acid sequences in the loop region where ϕC31 integrase is split are highlighted with a red box. (**B**) Structures of the split integrase-intein constructs. Changes made to the integrase sequence at the junctions where the fragments are fused to the split inteins are shown. The functional domains are shown as rectangular boxes: Integrase-**E^N^** (blue), *Npu* DnaE**^N^** (orange), *Ssp* DnaE**^C^** (purple) and Integrase-**E^C^** (green). Intein-catalysed protein *trans*-splicing generates an active version of the integrase in which residues at positions 308–312 of wild-type integrase (EGYRIQ) are replaced with EYCFN. The *trans*-spliced integrase is thus one amino acid shorter than the native integrase. (**C**) Variants of the split ϕC31 integrase designed to probe the requirements for reconstitution of integrase activity. (i) Integrase-EN; ϕC31 integrase (1–307). (ii) Integrase-**E^N^**-**I^N^**; Integrase-**E^N^** (residues 1–307) fused to the 102-amino acid residue *Npu* DnaE**^N^** (**I^N^**) using the two-residue EY and the N-terminal Cys residue from *Npu* DnaE**^N^**. (iii) Integrase-**E^N^**-I^N^*; ϕC31 integrase (1–307) fused to *Npu* DnaE**^N^** as described in (i) but with the EY residue at the splice site changed to GA to inactivate splicing activity. (iv) Integrase-EC; ϕC31 integrase C-extein (residues 314–605). (v) **I^C^**-Integrase-**E^C^**; ϕC31 integrase C-extein (residues 314–605) fused directly to the *Ssp* DnaE**^C^** (**I^C^**). *Ssp* DnaE**^C^** nucleophilic cysteine residue and the flanking residues required for splicing are highlighted (CFN). (vi) I^C*^-Integrase-**E^C^**; ϕC31 integrase C-extein fused to *Ssp* DnaE**^C^** as described in (i) but with the CFN residue at the splice site changed to ASA to inactivate splicing activity.

The codon-optimized ϕC31 integrase sequence was derived from pFM141 (24). Codon-optimized sequences of NpuN, a 102-amino acid residue split intein from *Nostoc punctiforme* DnaE and SspC, a 36-amino acid residue split intein from *Synechocystis sp*. DnaE, were from GeneArt (Invitrogen). Plasmids for constitutive expression of the N-terminal extein-intein fusion protein (Integrase-Ext**^N^**-I**^N^**) in *Escherichia coli* (Figure 2) were made by inserting protein-coding DNA sequences between NdeI and Acc65I sites in pMS140, a low-level expression vector with a pMB1 origin of replication (24). Plasmids for constitutive expression of the C-terminal intein–extein fusion protein (**I^C^**-Integrase-**E^C^**) were made in a similar way by inserting the coding sequence between NdeI and Acc65I sites in pEK76, which has a p15a (pACYC184) origin (24,25). Detailed properties of the vectors used here for expression of the recombinases have been described in detail elsewhere (9,24–27). Plasmids for *tet*-inducible expression of the split integrase components were made by cloning the protein-coding DNA sequences between SacI and SalI sites in pTet (28), whilst those for *ara*-inducible expression were made by cloning the protein-coding sequences between SacI and Sal sites in pBAD (29).

**Figure 3.**
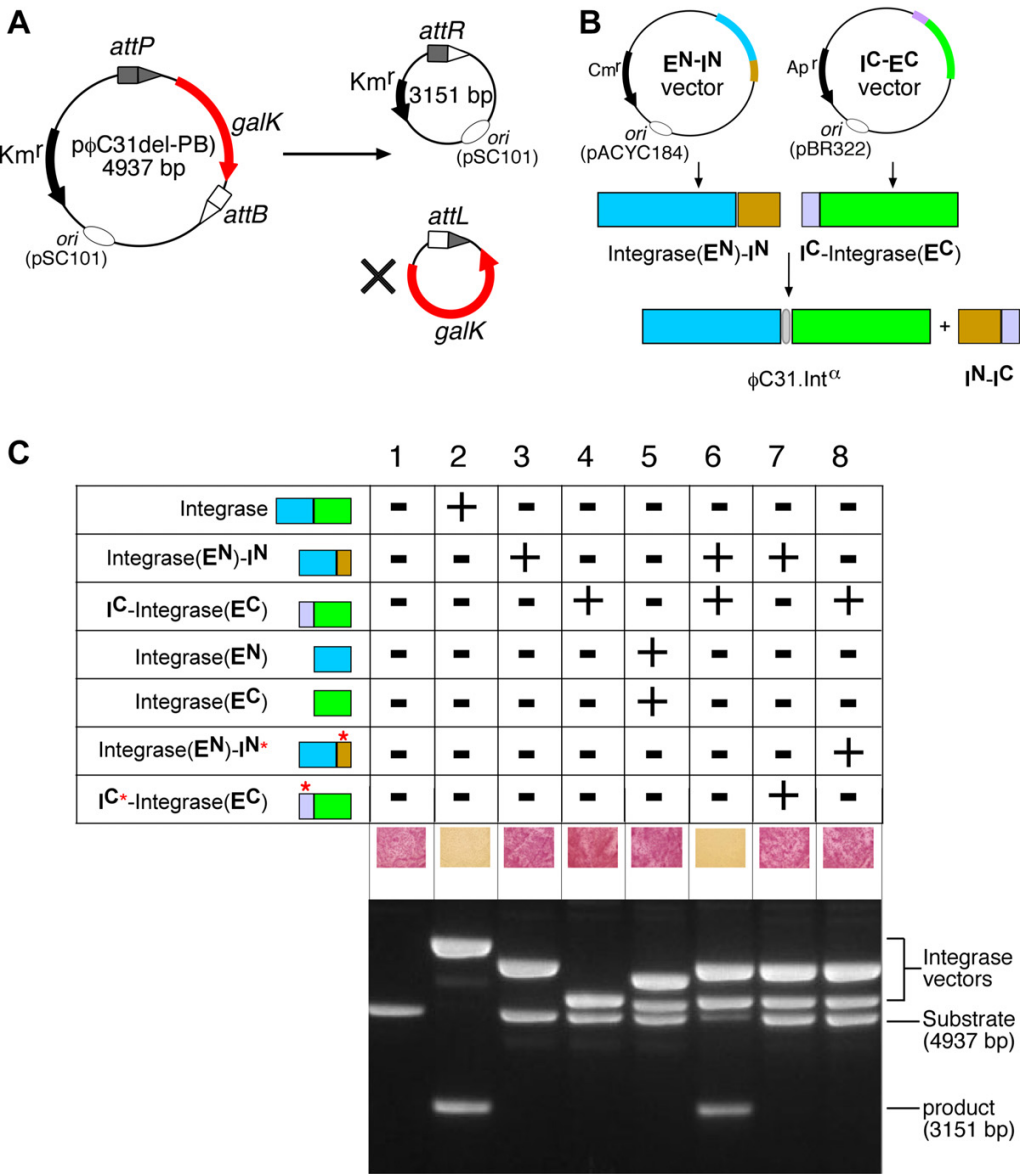
*In vivo* recombination activity of reconstituted split-intein ϕC31 integrase. (**A**) *In vivo* recombination assay using a deletion substrate (pϕC31-delPB) carrying the *galK* gene (24). The recombination product circle carrying the *galK* DNA has no origin of replication, and is lost during subsequent cell divisions. Inability of the cells to metabolize galactose leads to a change in colony colour on MacConkey galactose medium. (**B**) Constitutive expression of precursor polypeptides. The open reading frame of Integrase-**E^N^**-**I^N^** was expressed from a pACYC184-based vector, and **I^C^**-Integrase-**E^C^** was expressed from a pBR322-based vector (24). Expression of the two polypeptides followed by intein-catalysed protein *trans*-splicing reconstitutes ϕC31.Int**^α^**. (**C**) Assay of recombination activities (*galK* colour assay) of integrase constructs on *attP* × *attB* deletion substrates (pϕC31-delPB). In these assays, cells containing the substrate plasmid (pϕC31-delPB) were transformed with the expression vectors indicated and grown for 20 h in selection media. For analysis of *in vivo* recombination products, plasmid DNA was recovered from cells (24) and separated by means of 1.2% agarose gel electrophoresis. (1) Substrate only (blank control); (2) FEM141: ϕC31 Integrase; (3) FEM136: Integrase-**E^N^**-**I^N^**; (4) FEM137: **I^C^**-Integrase-**E^C^**; (5) FEM155 (Integrase-**E^N^**) and FEM157 (Integrase-**E^C^**); (6) FEM136 (Integrase-**E^N^**-**I^N^**) and FEM137 (**I^C^**-Integrase-**E^C^**); (7) FEM136 (Integrase-**E^N^**-**I^N^**) and FEM162 (I^C*^-Integrase-**E^C^**); (8) FEM137 (**I^C^**-Integrase-**E^C^**) and FEM161 (Integrase-**E^N^**-I^N^*).

The plasmid substrate for assessing recombination (deletion) by native ϕC31 integrase or *trans*-spliced ϕC31 integrase (pϕC31-delPB) was described in Olorunniji *et al.* (24). The sequences of ϕC31 *att* sites are shown in Supplementary Table S1. The test substrate contains a *galK* gene flanked by *attP* and *attB* sites arranged in direct repeat (head-to-tail orientation) resulting in deletion of the *galK* gene upon integrase-catalysed recombination. The plasmid substrate for assessing recombination (inversion) activity (pϕC31-invPB) was made by cloning the invertible promoter device shown in Figure 3A in a pSC101 origin plasmid with kanamycin resistance selection.

The DNA sequences of all protein constructs and plasmid substrates used in this study are shown in Supplementary Table S2.

### Recombination analysis

Assays for integrase-mediated recombination were carried out in *E. coli* strain DS941 (24). DS941 was first transformed with the test substrate plasmid (pϕC31-delPB or pϕC31-invPB). The substrate plasmid-containing strains were then either transformed with an integrase-expressing plasmid (pFM141), or co-transformed with two plasmids, each expressing part of the split integrase. The transformants were grown on selective MacConkey-galactose indicator plates (MacConkey agar base (Difco) supplemented with 1% galactose); kanamycin (50 μg/ml) was included to select for the substrate plasmid and the recombination product, whilst ampicillin (50 μg/ml) and/or chloramphenicol (25 μg/ml) were included to select for the integrase expression plasmids. Integrase-mediated deletion of the *galK* gene results in pale-coloured (*galK^−^*) colonies on the indicator plates, whereas red (*galK^+^*) colonies suggest lack of recombination. In some cases, colonies in which low-level recombination has occurred maintain a red colour. To determine the extent of recombination more accurately, plasmid DNA recovered from the cells was examined. L-broth (1 ml) was added to the surface of each plate and the cells were suspended using a plate spreader. An aliquot of this suspension was used to inoculate L-broth (1 in 1000 dilution), and the culture was incubated overnight at 37°C with kanamycin selection for the substrate and recombinant plasmids. Plasmid DNA was prepared using a Qiagen miniprep kit, and analysed by 1.2% agarose gel electrophoresis.

### Inducible expression of integrase-intein fusions for *trans*-splicing

*In vivo* expression of the integrase-intein fusion proteins under the control of arabinose and tet inducible promoters was carried out in strain DS941/Z1 (30), which consitutively expresses TetR, required for regulation of the pTet promoter. The DS941/Z1 strain was made competent by a standard calcium chloride method (31). The cells were transformed with the plasmid substrate pϕC31-invPB, then cultured for 90 min and selected on L-agar plates containing kanamycin (50 μg/ml). A single colony was picked and grown in kanamycin-containing L-broth (5 ml) to make a stationary phase overnight culture. An aliquot of the stationary phase culture was then diluted into L-broth containing kanamycin and grown to mid-log phase. These cells were made ‘chemically competent’ (as above) and transformed with the two vectors containing the coding sequences of the intein-integrase fragments. The plasmid vector pFEM148 (ampicillin selection) expresses Integrase-**E^N^**-**I^N^** under the control of the pTet promoter. The second plasmid pFEM149 (chloramphenicol selection) expresses **I^C^**-Integrase-**E^C^** under the control of the pBAD promoter.

The transformant cells were cultured for 90 min, and selected on L-agar plates containing kanamycin (50 μg/ml), ampicillin (100 μg/ml) and chloramphenicol (25 μg/ml). To carry out the recombination assays, a culture from a single colony was grown overnight in L-broth in the presence of the three antibiotics, to stationary phase. The culture was diluted further (1:100) and grown to mid-log phase (about 90 min), after which expression of the split-intein-integrase fragments was induced by the addition of anhydrotetracycline, aTc (0.1 μg/ml), arabinose, Ara (0.2% w/v) or both. Glucose was added to 0.4% concentration to the cultures where it is required to turn off expression of the arabinose promoter. The induced cultures were grown for 24 h at 37°C, after which the cultures were left at room temperature for 8 h. Next, aliquots of each culture (50 μl) were diluted with 950 μl phage buffer (10 mM Tris, pH 7.5, 10 mM MgCl_2_, 68 mM NaCl), and GFP expression was measured by means of fluorescence.

### Fluorescence measurements

Fluorescence measurements were carried out on a Typhoon FLA 9500 fluorimager (GE Healthcare). Aliquots of the diluted cultures (200 μl) were added to a 96-well plate, and the fluorescence of the expressed proteins was measured (GFP: excitation, 485 nm; emission, 520 nm and RFP: excitation, 532 nm; emission, 575 nm). To determine cell density for each sample, 50 μl aliquots were diluted to 1000 μl and the spectrophotometric absorbance was read at 600 nm. The GFP-independent background signals of the cells were determined by measuring the fluorescence of DS941/Z1 strain (containing the test substrate but without the split-intein-integrase expression vectors). The background fluorescence was subtracted from the values measured for samples in the different treatment groups, after normalization using the cell density measurements.

### Flow cytometry

Flow cytometry was used to measure single-cell fluorescence on a BD Accuri C6 instrument. Cells were washed in phosphate buffered saline and diluted to ∼10^6^ cells/ml. For each sample, GFP (λ_Ex_ 488 nm; λ_Em_ 533/30 nm) and RFP (λ_Ex_ 488 nm; λ_Em_ 585/40 nm) fluorescence was recorded. The forward scatter threshold was lowered to 10 000 to ensure acquisition of bacteria, and gating was performed to tightly select the dense population of bacteria depicted on a log scale plot of forward versus side scatter. Samples were acquired at a slow flow rate of 14 μl/min. Data were analysed using FlowJo™ software (Version 10.6.1).

## RESULTS AND DISCUSSION

### Design of split-intein serine integrase

We designed our split-intein system for conditional expression of ϕC31 integrase, a prototype serine integrase, based on previously reported systems (15). Since the natural DnaE split inteins used in this work require an invariant active site cysteine to remain in the spliced product protein (32–34), their use for split integrase reconstitution depends upon the identification of a short region of the protein sequence where insertion of a cysteine does not disrupt activity. In addition, for *trans*-splicing to be essential, the two extein components of the spliced integrase must not associate non-covalently to reconstitute a functional enzyme. We started by analysing the domain structure of serine integrases to determine where to split the protein, since a functional split intein could require introduction of mutations that would remain in the spliced protein product, if suitably placed natural Cys, Ser or Thr residues were unavailable. Based on sequence alignments of serine integrases (Figure 2A) and published crystal structures (35), we split ϕC31 integrase at the non-conserved region of the recombinase domain between the β9 and αI domains; this region includes a 10–12 residue loop which is absent in some related serine integrases (36) (Figure 2A). We therefore predicted that introduction of the required intein nucleophilic residue and any flanking residues would not have a deleterious effect on integrase activity. Furthermore, Lucet *et al.* (23) found that when the related large serine recombinase TnpX (from *Clostridium perfringens*) was split between the β9 and αI domains, the two fragments complemented each other to restore DNA binding (but not recombination) activity.

We then attached two well-characterised split-intein components to the ϕC31 integrase sequences; *Npu* DnaE**^N^**, **I^N^** (102 amino acids) from *Nostoc punctiforme* DnaE, and *Ssp* DnaE**^C^**, **I^C^** (36 amino acids) from *Synechocystis sp*. DnaE. The *Npu* DnaE**^N^** variant contains a L22S change and the *Ssp* DnaE**^C^** variant has a P21R mutation (32). This pair was chosen based on previous reports that the mutations confer high *trans*-splicing activity at 37°C in *E. coli* and tolerance to changes in amino acid sequence at the splicing junctions (15,32,37). *Npu* DnaE**^N^** (**I^N^**) was fused to the C-terminus of the N-terminal moiety of ϕC31 Integrase (Integrase-**E^N^**), and *Ssp* DnaE**^C^** (**I^C^**) was fused to the N-terminus of the C-terminal moiety of ϕC31 Integrase (Integrase-**E^C^**) (Figure 2B). Also, residues 308–310 of Integrase- **E^N^** were changed from EGY to EY, since these sequences exist naturally at the extein–intein junction of *Npu* DnaE**^N^**, and Integrase-**E^N^** residues 311–313 were changed from RIQ to CFN, sequences found at the intein-extein junction of *Ssp* DnaE**^C^** (32,37). It is known that these flanking residues are involved in enhancing the splicing efficiencies of this pair of split inteins (33,37). In the reconstituted *trans*-spliced integrase (ϕC31.Int**^α^**), the natural 6-residue sequence at positions 308 to 313, EGYRIQ, is replaced with the 5-residue sequence EYCFN. We made these changes to maximize the efficiency of the splicing reaction in order to optimize activity in *E. coli*. Hence the reconstituted *trans*-spliced integrase, ϕC31.Int**^α^** is one amino acid residue shorter than the wild-type ϕC31 integrase. Since these changes are in a non-conserved region (see above), we predicted that they would not substantially affect recombination activity.

### Intein-mediated reconstitution of functional ϕC31 integrase

To assay *in vivo* recombination activity of our split-intein ϕC31 integrase, we used a well-characterized colour-based *galk* assay (Figure 3A; 26,27,38). The *attP* and *attB* recombination sites (Supplementary Table S1) on the substrate plasmid pϕC31-delPB are in a direct repeat orientation, so that recombination between them causes deletion of the *galK* gene. Pale-coloured (*galK*−) colonies on the indicator plates indicate recombination proficiency, whereas red (*galK+*) colonies indicate incomplete or zero recombination. The coding sequences for Integrase-**E^N^**-**I^N^** and **I^C^**-Integrase-**E^C^** (and also appropriate control proteins; see Figure 2C) were cloned into separate low-level expression vectors (24), as illustrated in Figure 3B. An *E. coli* strain containing the test substrate was transformed with these plasmids, and recombination activity was assessed by colony colour and by gel electrophoresis analysis of plasmid DNA recovered from the cells (Figure 3C).

Neither Integrase-**E^N^**-**I^N^** (Figure 3C, lane 3) nor **I^C^**-Integrase-**E^C^** (Figure 3C, lane 4) on their own were able to catalyse *attP* x *attB* recombination. Furthermore, co-expression of the N-extein (Integrase-**E^N^**) and C-extein (Integrase-**E^C^**) components of the integrase (lane 5) gave no recombination. This important control shows that the intein-less precursor proteins Integrase-**E^N^** and Integrase-**E^C^** do not complement each other by non-covalent association to give recombination activity. This contrasts with reported split tyrosine recombinases, the components of which associate to reconstitute recombination activity without protein splicing (18–20). When the intein-tagged integrase exteins Integrase-**E^N^**-**I^N^** and **I^C^**-Integrase-**E^C^** were co-expressed, reconstitution of integrase recombination activity was observed (lane 6). The DNA analysis shown in the lower panel of Figure 3c (lanes 1–6) suggests that recombination by the trans-spliced integrase (ϕC31.Int**^α^**) has proceeded to over 80%.

Our results show that the changes from the wild-type integrase sequence that had to be introduced at the splice site are compatible with recombination activity. As many other serine integrases have similarly non-conserved, variable lengths of amino acid sequence in the region of the protein between β9 and αI (Figure 2A), we predict that these enzymes could also be engineered to create active split-intein systems.

### *Trans*-splicing is required for integrase recombination activity

The results shown in Figure 3C show that the *Npu* DnaE and *Ssp* DnaE intein moieties are required for reconstitution of recombination activity, but these experiments do not unambiguously establish the requirement for *trans*-splicing. Some split inteins are known to associate tightly via non-covalent interactions (39), and this can lead to reconstitution of the split protein activity, without splicing (40). To test whether this applies in our split integrase system, we mutated the nucleophilic cysteine residue and rate-enhancing flanking residues at the active sites of the two intein moieties (29) to render them catalytically inactive. We changed the junction residues ‘EY’ in the Integrase-**E^N^** moiety to ‘GA’ and the Cys residue in the *Npu* DnaE**^N^** moiety of Integrase-**E^N^**-**I^N^** to ‘A’ to give the mutated version Integrase-**E^N^**-I^N^* (Figure 2c). Similarly, the residues ‘CFN’ in the *Ssp* DnaE**^C^** moiety of **I^C^**-Integrase-**E^C^** were changed to ‘ASA’, to derive the mutated version I^C*^-Integrase-**E^C^** (Figure 2C). No recombination activity was observed when active Integrase-**E^N^**-**I^N^** was co-expressed with inactive I^C*^-Integrase-**E^C^** (Figure 3C, lane 7), nor when active **I^C^**-Integrase-**E^C^** was co-expressed with inactive Integrase-**E^N^**-I^N^* (Figure 3C, lane 8), showing that reconstitution of recombination activity requires the catalytic activities of both split intein fragments. In this aspect, serine integrases differ from integrases of the tyrosine recombinase family where non-covalent association is sufficient to allow reconstitution of activity from split protein fragments (18–20).

The *attP* x *attB in vivo* deletion reactions were slower in cultures where recombination required post-translational *trans*-splicing to reconstitute functional integrase, when compared to recombination by native integrase (see Supplementary Figure S1). It is therefore likely that the rate of *trans*-splicing between *Npu* DnaE**^N^** and *Ssp* DnaE**^C^** in our cell cultures is significantly slower than the fast splicing reaction rate observed *in vitro* for these intein pairs (37). The reduced rate might be due to limiting amounts of the two precursor polypeptides expressed in the cells. It is possible that this delay is caused by the different rates of expression of the integrase fragments from the two different expression vectors used in this study, rather than the actual *trans*-splicing reaction *in vivo*.

### Split intein-regulated integrase-catalysed inversion system

**Figure 4.**
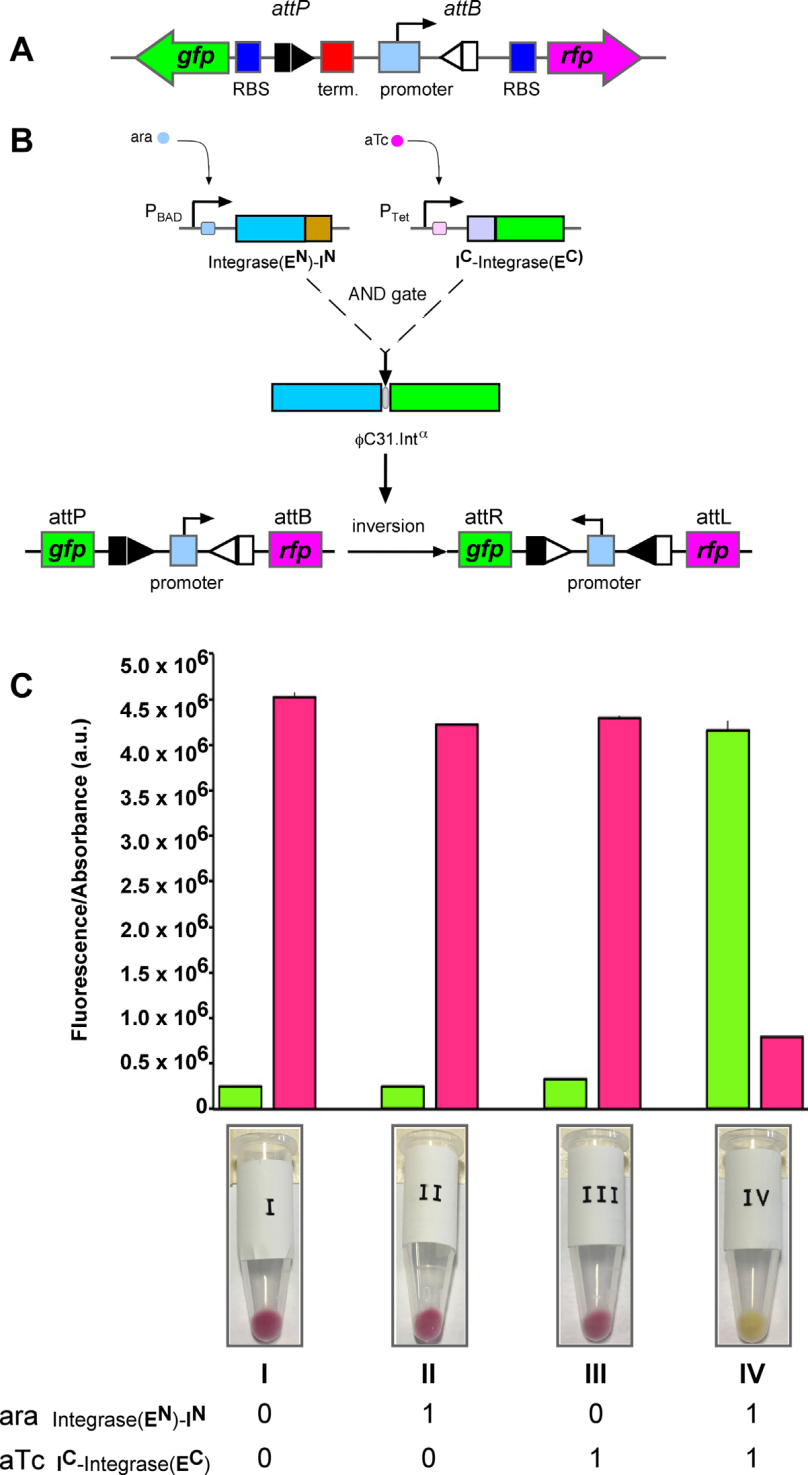
Controlling the function of an invertible promoter system using split intein-regulated ϕC31 integrase activity. (**A**) Design of a recombinase-operated switch using an invertible promoter reporter system. The constitutive promoter sequence (cyan rectangle; Biobrick J23104) is flanked by ϕC31 integrase *attP* and *attB* sites (grey arrows) that are arranged in inverted orientation. In its default state, the promoter constitutively drives the expression of a red fluorescent protein (*rfp*) gene (pink arrow). The T1 terminator sequence (red squares) located immediately upstream of the promoter prevents transcriptional read-through to the green fluorescent protein (*gfp*) gene (green arrow). Upon integrase-catalysed site-specific recombination, the orientation of the promoter is reversed to allow the expression of GFP and block RFP production. A Biobrick ribosomal binding site, RBS, (RBS_B0034, blue rectangles), is positioned 5′ of the *rfp* and *gfp* genes to drive optimal translation of the synthesized mRNAs. (**B**) Conditional expression of ϕC31 integrase fragments and split intein-mediated reconstitution of activity. The two split-intein integrase fragments are expressed from two inducible promoter vectors. Arabinose (ara) induces expression of Integrase-**E^N^**-**I^N^** under the control of the pBAD promoter (pFEM149), whilst anhydrotetracycline (aTc) induces expression of **I^C^**-Integrase-**E^C^** under the control of the pTet promoter (pFEM148). Post-translational *trans*-splicing of the split-intein integrase fragments generates the functional reconstituted integrase (ϕC31.Int**^α^**). Catalysis of *attP* x *attB* inversion by ϕC31.Int**^α^** results in reversal of the orientation of the promoter. (**C**) Validation of the split-intein ϕC31 integrase recombinational AND-gate. Fluorescence measurements indicating the expression of GFP and RFP in cells induced with aTc and/or ara. Cells were grown at 37°C for 24 h in the presence of aTc (0.1 μg/ml) and ara (0.2%). Glucose was added to 0.4% concentration to treatments A and C to turn off expression of the arabinose promoter. Each bar represents mean and standard deviation of four determinations. Below the bar chart are images of Eppendorf tubes showing the pellet obtained after centrifugation of 5 ml of bacterial culture.

To demonstrate the potential application of our split intein ϕC31 integrase in conditional expression of recombination activity, we designed an invertible genetic system based on an inversion substrate plasmid, pϕC31-invPB. Recombination between *attP* and *attB* sites in pϕC31-invPB inverts the orientation of a constitutive promoter sequence (Biobrick J23104), thereby switching expression from RFP to GFP (Figure 4A). The *E. coli* strain DS941/Z1/pϕC31-invPB was co-transformed with two vectors, each expressing one of the two split-intein integrase fragments. Expression of Integrase-**E^N^**-**I^N^** was placed under the control of the *P_BAD_* promoter, and expression of **I^C^**-Integrase-**E^C^** was placed under the control of the P*tet* promoter (Figure 4B). Co-expression of the split-intein fragments (and thus recombination) is dependent on the presence of both of the inducers arabinose and anhydrotetracycline (aTc) (Figure 4C). No recombination (GFP expression) was observed unless both split-intein components were expressed (when aTc and arabinose were added to the growth medium; column IV.

The split-intein regulated serine integrase can be deployed as an effective tool for transient inversion of genetic regulatory modules (Figure 4). The ability to reconstitute integrase activity from inactive protein fragments using split inteins could allow faster control of site-specific recombination by specific activation of intein splicing. This could be achieved by means of light-sensitive protein domains that regulate intein splicing (39,40) or small molecule ligands that act directly on the split inteins (41) to control the protein-protein association step that precedes protein *trans*-splicing. Fast, accurate switching on of enzyme activity *in vivo* could be achieved if methods for efficient conditional activation of protein *trans*-splicing were available (42). An engineered photo-activatable gp41-1 split intein system was recently shown to work in bacterial cells (43), demonstrating the feasibility of this approach. Others have demonstrated the use of pH changes (44) and temperature (45) as tools for regulating intein functions *in vitro*. In our system, conditional splicing would enable fast activation of recombination by reconstituting integrase from the precursors which are already present. Further development of these technologies can enhance capacity for building orthogonal logic gate components for conditional gene expression regulation and genome engineering applications, and add to the existing tools for programmable cellular functions (46,47).

### Intein-mediated assembly of integrase-RDF fusion recombinase and catalysis of *attR* x *attL* recombination

**Figure 5.**
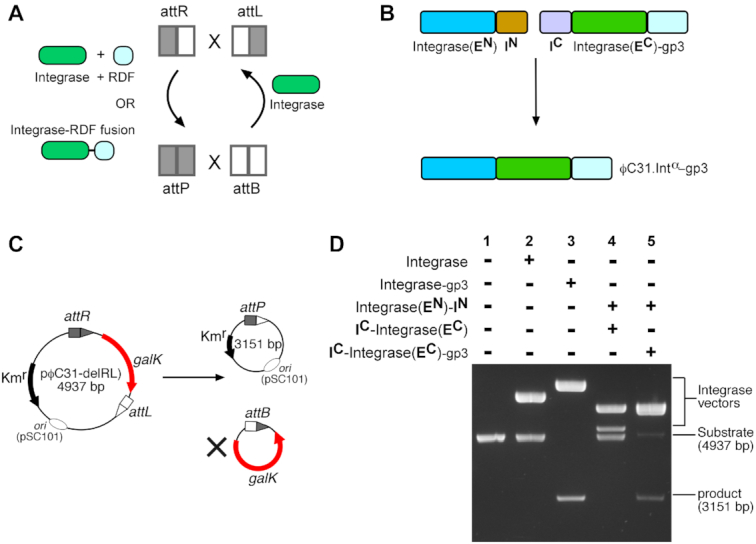
Intein-mediated assembly of ϕC31 integrase-RDF fusion recombinase and catalysis of *attR* x *attL* recombination. (**A**) Catalysis of *attR* x *attL* recombination by integrase + RDF, or integrase-RDF fusion protein (see text for details). The RDF for ϕC31 integrase is gp3. (**B**) Split-intein catalysed assembly of integrase-RDF fusion recombinase. Non-covalent association of Integrase-**E^N^**-**I^N^** and **I^C^**-Integrase-**E^C^**-gp3 via the intein domains (**I^N^** and **I^C^**) is followed by a *trans*-splicing reaction that gives the fusion recombinase ϕC31.Int**^α^**-gp3. (**C**) Assay of recombination activities (*galK* colour assay) of integrase constructs on *attR* × *attL* deletion substrate (pϕC31-delRL). The test substrate is identical to pϕC31-delPB (Figure 3A), except that the *attP* and *attB* sites are replaced by *attR* and *attL* respectively. (**D**) The *in vivo* recombination assay and analysis are as described in Figure 4C. (1) Substrate only (blank control); (2) FEM141: ϕC31.Integrase; (3) FEM33: ϕC31.Integrase-gp3 fusion; (4) FEM136 (Integrase-**E^N^**-**I^N^**) and FEM137 (**I^C^**-Integrase-**E^C^**); (5) FEM136 (Integrase-**E^N^**-**I^N^**) and FEM188 (**I^C^**-Integrase-**E^C^**-gp3).

We recently showed that recombination between *attR* x *attL* sites can be catalysed efficiently by artificial proteins in which the recombination directionality factor (RDF) is fused to the integrase using a short peptide linker. The fusion recombinase ϕC31.integrase-gp3 catalysed *attR* x *attL* recombination efficiently (24) (Figure 5A). This system allows more predictable regulation of integrase-catalysed conversion of *attP*/*attB* to *attR*/*attL* and vice versa, with potential applications in building genetic switches and recombinase-based counters.

To demonstrate the use of our split-intein regulated system in *attR* x *attL* recombination, we split ϕC31.integrase-gp3 between the β9 and αI domains of the integrase (Figure 5B), at the same position as described above for ϕC31 integrase itself. To test for recombination activity, we used a test substrate (pϕC31-delRL) similar to the *attP* x *attB* substrate (pϕC31-delPB) but with the *attP* and *attB* sites replaced by *attR* and *attL*, respectively (Supplementary Table S1). The sites are arranged in the head-to-tail orientation such that deletion of the *galK* gene leads to a reaction product that is smaller in size than the starting substrate (Figure 5C). As expected, neither integrase (Figure 5D, lane 2) nor co-expression of the Integrase-**E^N^**-**I^N^** and **I^C^**-Integrase-**E^C^** (lane 4) were able to catalyse *attR* x *attL* recombination. In contrast, co-expression of the intein-tagged integrase N-extein (Integrase-**E^N^**-**I^N^**) and C-extein-gp3 (**I^C^**-Integrase-**E^C^**-gp3) results in efficient *attR* x *attL* recombination (lane 5). The extent of recombination by the *trans*-spliced fusion recombinase (ϕC31.Int**^α^**-gp3) was lower than that catalysed by native ϕC31.integrase-gp3 (lane 3), in line with our observations for wild-type integrase and its *trans*-spliced analogue (see above).

### Time courses of recombination by wild-type and *trans*-spliced integrase

**Figure 6.**
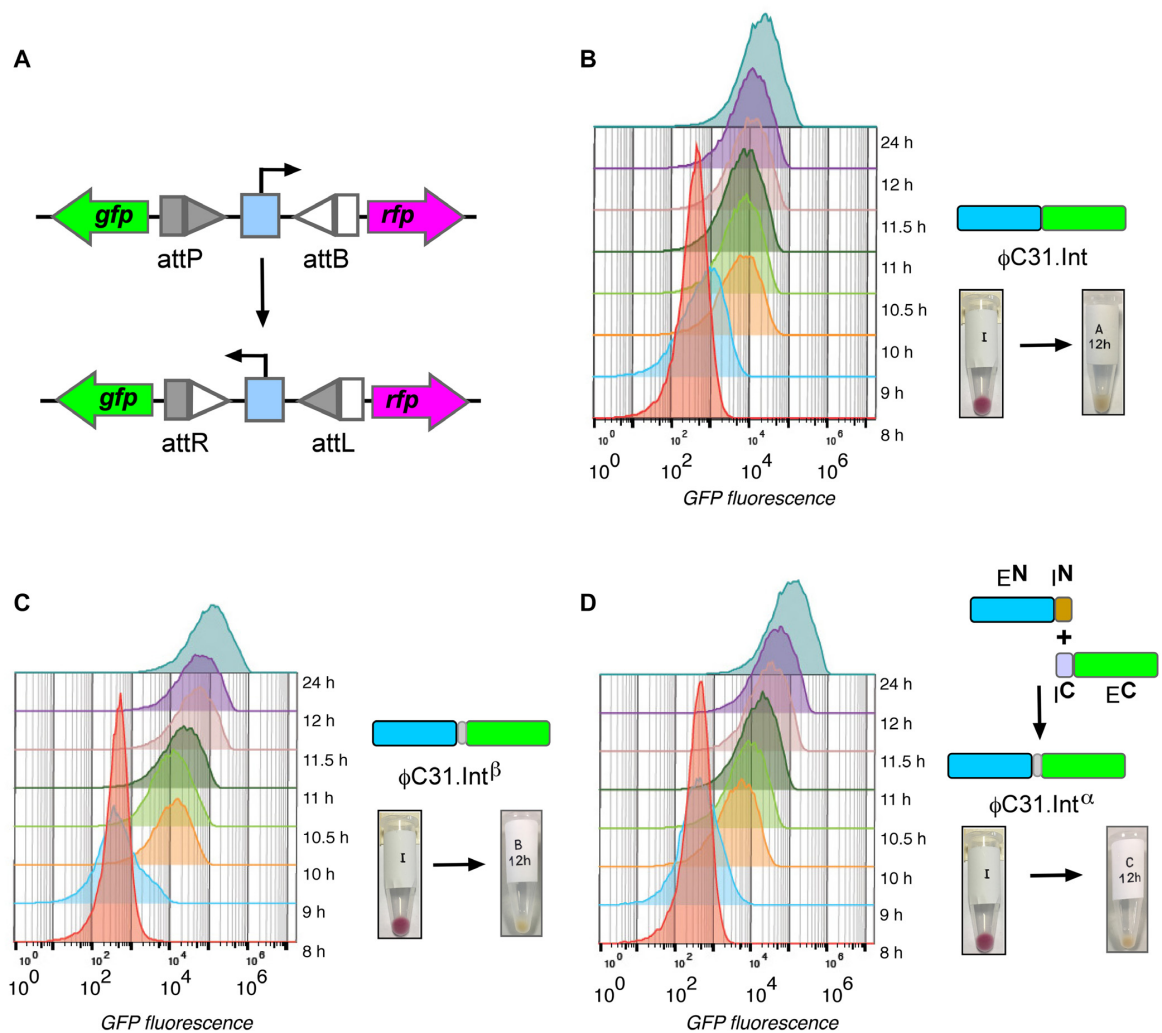
Cytometry time course assays of *attP* x *attB* recombination. (**A**) The assays were carried out using the invertible promoter system described in Figure 4, in which integrase-catalysed inversion switches expression from RFP to GFP. (**B**) Wild-type ϕC31 integrase. (**C**) Full length ϕC31 integrase with ‘EYCFN’ mutations (ϕC31.Int^β^). (**D**) *Trans*-spliced ϕC31 integrase formed *in situ* (ϕC31.Int**^α^**). Integrases were expressed from appropriate plasmid vectors as follows: (B) ϕC31 Integrase, pFEM141; (C) EYCFN ϕC31 Integrase, pFEM204; (D) Splicing precursor proteins Integrase-**E^N^**-**I^N^**, pFEM136 and **I^C^**-Integrase-**E^C^**, pFEM137. The extent of recombination at the indicated time points was monitored by measuring the amount of GFP formed.

To characterize further the effects of the EYCFN mutations introduced into the reconstituted integrase (ϕC31.Int**^α^**) after *trans*-splicing, we carried out a time course comparison of recombination catalysed by reconstituted and wild-type integrase. To do this, we made a plasmid expressing the expected product of *trans*-splicing, with the EYRIQ sequence in wild-type ϕC31 integrase replaced with EYCFN. We refer to this construct as ϕC31.Int**^β^**. Recombination activity was measured using the invertible promoter reporter system (see Figure 4). The extent of recombination was monitored by measuring the progress of switching from RFP to GFP expression, using single-cell flow cytometry (Figure 6A). *Escherichia coli* DS941 cells containing the pϕC31-invPB substrate were transformed with appropriate vector plasmids expressing wild-type ϕC31 integrase, ϕC31.Int (Figure 6B); full length ϕC31 integrase with the EYCFN mutations, ϕC31.Int^β^ (Figure 6C); and reconstituted *trans*-spliced ϕC31 integrase, ϕC31.Int^α^ (Figure 6D). Cells were cultured with appropriate antibiotic selection and sampled at 8, 9, 10, 10.5, 11, 11.5, 12 and 24 h. The expression of GFP following recombination by wild-type integrase was observable early and peaked at about 10.5 h; a slight further increase in GFP expression was observed at 24 h (Figure 6B). The progress of the reaction by the ϕC31 integrase with the EYCFN mutations, ϕC31.Int^β^, was slightly slower, showing a steady increase in GFP expression from 9 to 11.5 h and an even higher level of expression after 24 h. The mutations thus caused a slight decrease in integrase activity (Figure 6C). In contrast, increase in GFP expression was noticeably slower when protein *trans*-splicing (ϕC31.Int^β^) was required to reconstitute the integrase and activate recombination activity (Figure 6D). This is consistent with our interpretation of the lower activity of *trans*-spliced integrase in the experiment shown in Figure 3.

## CONCLUSION

Serine integrases promote efficient directional DNA site-specific recombination, and thus have major potential applications in genome engineering and metabolic pathway engineering (9,11,48–51). They have also been incorporated into the design of cellular state machines and biocomputing devices (4,8,11). Here we have demonstrated a novel method for regulation of serine integrase activity by making activity dependent on post-translational *trans-*splicing of two integrase extein components. Our experiments demonstrate that the split-intein regulated recombination system can potentially be used to toggle between two DNA states in which the forward reaction is catalysed by a serine integrase, and the reverse reaction by an integrase-RDF fusion. In a practical application, reconstitution of the integrase and the integrase-RDF fusion could be mediated by two pairs of orthogonal split inteins. Several types of split inteins have been described in the literature (52,53), and further characterization of their properties would make them available as orthogonal functional parts. Recently, mutations of the *Npu* DnaE split intein have been identified that affect the specificity, reaction rates, and tolerance to changes in the flanking residues (16,17). Use of these intein variants that tolerate a wider range of amino acid residues around the splice site would require fewer changes to the extein sequence of the target integrase, thereby reducing the potential risk of introducing deleterious mutations and making it easier to find suitable split positions.

## Supplementary Material

gkz936_Supplemental_File
