# Supplementary material for: Control of ϕC31 integrase-mediated site-specific recombination by protein trans-splicing
Source: Nucleic Acids Res. 2019 Oct 31;47(21):11452–60. doi: 10.1093/nar/gkz936 (PMC6868429; doi:10.1093/nar/gkz936)
Supplement: gkz936_Supplemental_File [file gkz936_supplemental_file.pdf]

SUPPLEMENTARY DATA

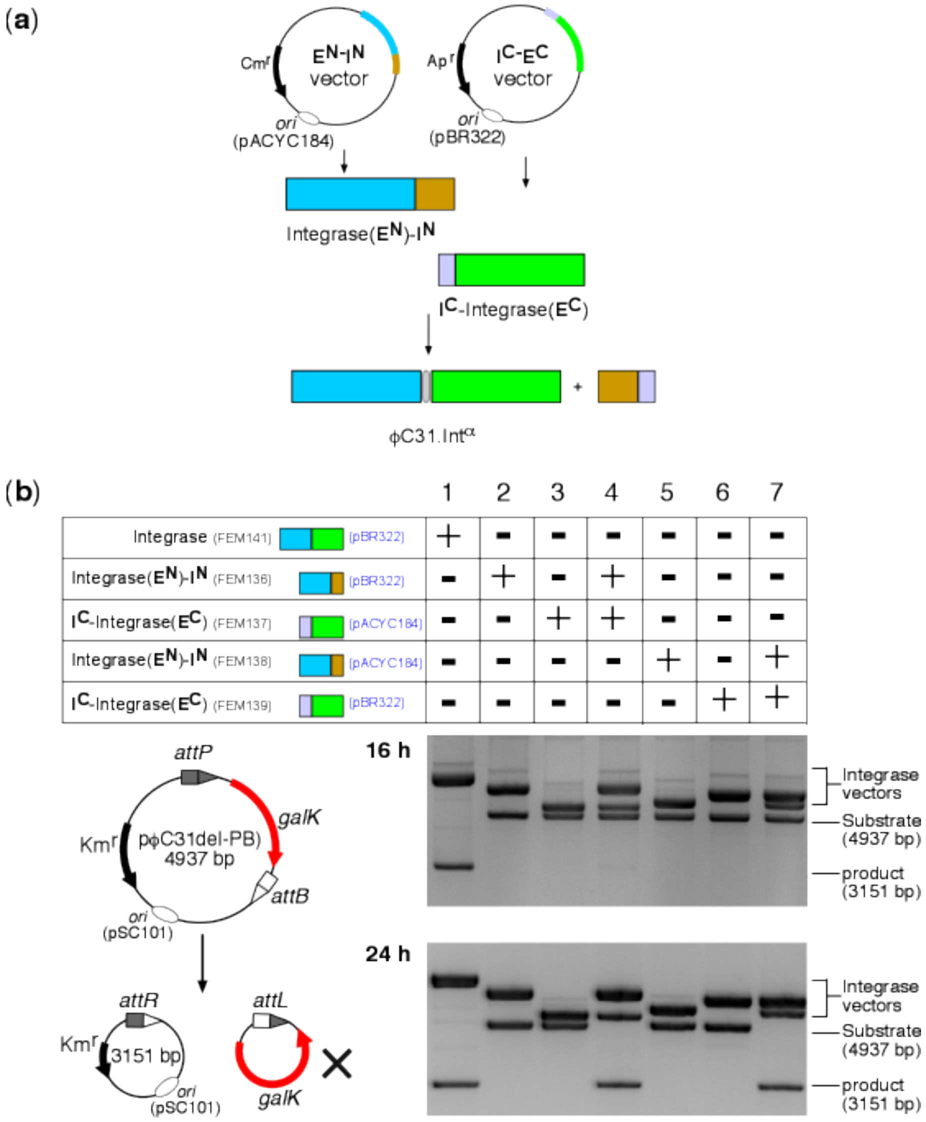

Figure S1: Comparison of *in vivo* recombination activity of native ϕC31 integrase with reconstituted trans-spliced ϕC31 integrase

(A) The precursor polypeptides for the split-intein integrase were consitutively expressed from vectors with either a pACYC184 or pBR322 origin of replication (Proudfoot *et al.*, 2011). For each precursor, both types of vector were tested, to confirm that the expression vector did not have any serious effect on the outcome of the experiment (see part B). In the example shown, the **E^N –I^N** vector is expressed in pACY184, and the **I^C-E^C** construct placed in pBR322 vector. (**B**) Assay of recombination activities (*galK* deletion assay) of integrase constructs on an *attP* × *attB* substrate (pϕC31-delPB). The *in vivo* recombination assay and analysis are as described in Figure 4C. Cells containing pϕC31-delPB were transformed with the expression vectors indicated and grown for 16 hours (top panel, **16 h**) or for 24 hours (bottom panel, **24 h**) on selective plates. For analysis of *in vivo* recombination products, plasmid DNA was recovered from cells (Olorunniji *et al.*, 2017) and separated by means of 1.2% agarose gel electrophoresis.

Table S1

Sequences of ΦC31 integrase att sites. The sequences are shown in the head-to-tail orientation that results in deletion of the intervening sequences upon recombination. The central 2-bp overlap sequences of the att sites are highlighted in bold text.

ΦC31 (*attP*)
AGTAGTGCCCCAACTGGGGTAACCT**TT**GAGTTCTCTCAGTTGGGGGCGTA
TCATCACGGGGTTGACCCCATTGGA**AA**CTCAAGAGAGTCAACCCCCGCAT

ΦC31 (*attB*)
GCGGTGCGGGTGCCAGGGCGTGCCC**TT**GGGCTCCCCGGGCGCGTACTCC
CGCCACGCCCACGGTCCCGCACGGG**AA**CCCGAGGGGCCCGCGCATGAGG

ΦC31 (attR)
AGTGCCCCAACTGGGGTAACCT**TT**GGGCTCCCCGGGCGCGTACTCCACC
TCACGGGGTTGACCCCATTGGA**AA**CCCGAGGGGCCCGCGCATGAGGTGG

ΦC31 (*attL*)
GCGGTGCGGGTGCCAGGGCGTGCCC**TT**GAGTTCTCTCAGTTGGGGGCGTA
CGCCACGCCCACGGTCCCGCACGGG**AA**CTCAAGAGAGTCAACCCCCGCAT

Table S2: Plasmid DNA sequences of protein constructs (integrases, integrase-RDF fusion, split integrase fragments, split inteins) and plasmid substrates used in this study.

(a) *In vivo* expression vectors derived from pBR322.

The features of pFEM141 and pFEM33 (both derived from pMS140 and sequences shown below) have been described in detail in Olorunniji *et al*., (2017). In these plasmids, integrase ORFs are cloned between NdeI and Acc65I sites, except pFEM33 in which NdeI and XhoI were used due to the presence of an internal Acc65I site in the ORF of the integrase-RDF fusion construct.

pFEM141 φC31.Integrase expression plasmid DNA sequence

GAATTCTAGAAATAATTTTGTTTAACTTTAAGAAGGAGATATA**CATATG**GATACCTATGCCGGTGCCTATGATCGTCA
GAGCCGTGAACGTGAAAATAGCAGCGCAGCAAGTCCGGCAACCCAGCGTAGCGCAAATGAAGATAAAGCAGCCGATCT
GCAGCGTGAAGTTGAACGTGATGGTGGTCGTTTTCGTTTTGTTGGTCATTTTAGCGAAGCACCGGGTACAAGCGCATT
TGGCACCGCAGAACGTCCGGAATTTGAACGTATTCTGAATGAATGTCGTGCAGGTCGTCTGAACATGATTATTGTTTA
TGATGTGAGCCGTTTTAGCCGTCTGAAAGTTATGGATGCAATTCCGATTGTTAGCGAACTGCTGGCACTGGGTGTTAC
CATTGTTAGCACCCAAGAAGGTGTTTTTCGTCAGGGTAATGTTATGGATCTGATTCATCTGATTATGCGTCTGGATGC
AAGCCATAAAGAAAGCAGCCTGAAAAGCGCAAAAATCCTGGATACCAAAAACCTGCAGCGCGAACTGGGTGGTTATGT
TGGTGGTAAAGCACCGTATGGTTTTGAACTGGTTAGCGAAACCAAAGAAATCACCCGTAATGGTCGTATGGTTAACGT
GGTTATTAACAAACTGGCACATAGCACCACACCGCTGACCGGTCCGTTTGAATTTGAGCCGGATGTTATTCGTTGGTG
GTGGCGTGAAATCAAAACCCATAAACATCTGCCGTTTAAACCGGGTAGCCAGGCAGCAATTCATCCGGGTAGCATTAC
CGGTCTGTGTAAACGTATGGATGCCGATGCAGTTCCGACCCGTGGTGAAACCATTGGTAAAAAAACCGCAAGCAGCGC
ATGGGACCCGGCAACAGTTATGCGTATTCTGCGTGATCCGCGTATTGCCGGTTTTGCAGCAGAAGTGATCTACAAAAA
AAAACCTGATGGCACCCCGACCACCAAAATTGAAGGTTATCGTATCCAGCGCGATCCGATTACCCTGCGTCCGGTTGA
ACTGGATTGTGGTCCGATTATTGAACCGGCAGAATGGTATGAACTGCAGGCATGGCTGGATGGTCGTGGTCGCGGTAA
AGGTCTGAGCCGTGGTCAGGCAATTCTGAGCGCAATGGATAAACTGTATTGTGAATGTGGTGCCGTTATGACCAGCAA
ACGTGGTGAAGAAAGCATCAAAGATAGTTATCGTTGTCGTCGTCGTAAAGTTGTTGATCCGAGCGCACCGGGTCAGCA
TGAAGGCACCTGTAATGTTAGCATGGCAGCACTGGATAAATTTGTTGCCGAACGCATCTTTAACAAAATTCGTCATGC
CGAAGGTGATGAAGAAACCCTGGCACTGCTGTGGGAAGCAGCACGTCGTTTTGGTAAACTGACCGAAGCTCCGGAAAA
AAGCGGTGAACGTGCCAATCTGGTTGCAGAACGTGCAGATGCACTGAATGCACTGGAAGAACTGTATGAAGATCGTGC
AGCGGGTGCGTATGATGGTCCGGTTGGTCGTAAACATTTTCGTAAACAGCAGGCAGCCCTGACCCTGCGCCAGCAGGG
TGCAGAAGAACGTCTGGCAGAACTGGAAGCCGCAGAAGCACCGAAACTGCCGCTGGATCAGTGGTTTCCGGAAGATGC
AGATGCCGATCCGACAGGTCCGAAAAGTTGGTGGGGTCGTGCAAGCGTTGATGATAAACGTGTTTTTGTGGGTCTGTT
CGTGGATAAAATTGTGGTTACCAAAAGCACCACCGGTCGCGGTCAGGGTACACCGATTGAAAAACGTGCAAGCATTAC
CTGGGCAAAACCGCCTACCGATGATGATGAAGATGATGCACAGGATGGCACCGAAGATGTTGCAGCAACTAGTCACCA
TCACCATCACCATTAATAA**GGTACC**TCTAGAGCTTGAGTATTCTATAGTGTCACCTAAATAGCTTGGCGTAATCATGG
TCATAGCTGTTTCCTGTGTGAAATTGTTATCCGCTCACAATTCCACACAACATACGAGCCGGAAGCATAAAGTGTAAA
GCCTGGGGTGCCTAATGAGTGAGCTAACTCACATTAATTGCGTTGCGCTCACTGCCCGCTTTCCAGTCGGGAAACCTG
TCGTGCCAGCTGCATTAATGAATCGGCCAACGCGCGGGGAGAGGCGGTTTGCGTATTGGGCGCTCTTCCGCTTCCTCG
CTCACTGACTCGCTGCGCTCGGTCGTTCGGCTGCGGCGAGCGGTATCAGCTCACTCAAAGGCGGTAATACGGTTATCC
ACAGAATCAGGGGATAACGCAGGAAAGAACATGAATTAATTCTCATGTTTGACAGCTTATCATaGATTAGCTTTAATG
CGGTAGTTTATCACAGTTAAATTGCTAACGCAGTCAGGCACCGTGTATGAAATCTAACAATGCGCTCATCGTCATCCT
CGGCACCGTCACCCTGGATGCTGTAGGCATAGGCTTGGTTATGCCGGTACTGCCGGGCCTCTTGCGGGATCGACGCGA
GGCTGGATGGCCTTCCCCATTATGATTCTTCTCGCTTCCGGCGGCATCGGGATGCCCGCGTTGCAGGCCATGCTGTCC
AGGCAGGTAGATGACGACCATCAGGGACAGCTTCAAGGATCGCTCGCGGCTCTTACCAGCCTAACTTCGATCACTGGA
CCGCTGATCGTCACGGCGATTTATGCCGCCTCGGCGAGCACATGGAACGGGTTGGCATGGATTGTAGGCGCCGCCCTA
TACCTTGTCTGCCTCCCCGCGTTGCGTCGCGGTGCATGGAGCCGGGCCACCTCGACCTGAATGGAAGCCGGCGGCACC
TCGCTAACGGATTCACCACTCCAAGAATTGGAGCCAATCAATTCTTGCGGAGAACTGTGAATGCGCAAACCAACCCTT
GGCAGAACATATCCATCGCGTCCGCCATCTCCAGCAGCCGCACGCGGCGCATCTCGGGCAGCGTTGGGTCCTGGCCAC
GGGTGCGCATGATCGTGCTCCTGTCGTTGAGGACCCGGCTAGGCTGGCGGGGTTGCCTTACTGGTTAGCAGAATGAAT
CACCGATACGCGAGCGAACGTGAAGCGACTGCTGCTGCAAAACGTCTGCGACCTGAGCAACAACATGAATGGTCTTCG
GTTTCCGTGTTTCGTAAAGTCTGGAAACGCGGAAGTCAGCGCCCTGCACCATTATGTTCCGGATCTGCATCGCAGGAT
GCTGCTGGCTACCCTGTGGAACACCTACATCTGTATTAACGAAGCGCTGGCATTGACCCTGAGTGATTTTTCTCTGGT
CCCGCCGCATCCATACCGCCAGTTGTTTACCCTCACAACGTTCCAGTAACCGGGCATGTTCATCATCAGTAACCCGTA
TCGTGAGCATCCTCTCTCGTTTCATCGGTATCATTACCCCCATGAACAGAAATTCCCCCTTACACGGAGGCATCAAGT
GACCAAACAGGAAAAAACCGCCCTTAACATGGCCCGCTTTATCAGAAGCCAGACATTAACGCTTCTGGAGAAACTCAA
CGAGCTGGACGCGGATGAACAGGCAGACATCTGTGAATCGCTTCACGACCACGCTGATGAGCTTTACCGCAGCTGCCT
CGCGCGTTTCGGTGATGACGGTGAAAACCTCTGACACATGCAGCTCCCGGAGACGGTCACAGCTTGTCTGTAAGCGGA
TGCCGGGAGCAGACAAGCCCGTCAGGGCGCGTCAGCGGGTGTTGGCGGGTGTCGGGGCGCAGCCATGACCCAGTCACG
TAGCGATAGCGGAGTGTATACTGGCTTAACTATGCGGCATCAGAGCAGATTGTACTGAGAGTGCACCATatATGCGGT

GTGAAATACCGCACAGATGCGTAAGGAGAAAATACCGCATCAGGCGCTCTTCCGCTTCCTCGCTCACTGACTCGCTGC
GCTCGGTCGTTCGGCTGCGGCGAGCGGTATCAGCTCACTCAAAGGCGGTAATACGGTTATCCACAGAATCAGGGGATA
ACGCAGGAAAGAACATGTGAGCAAAAGGCCAGCAAAAGGCCAGGAACCGTAAAAAGGCCGCGTTGCTGGCGTTTTTCC
ATAGGCTCCGCCCCCCTGACGAGCATCACAAAAATCGACGCTCAAGTCAGAGGTGGCGAAACCCGACAGGACTATAAA
GATACCAGGCGTTTCCCCCTGGAAGCTCCCTCGTGCGCTCTCCTGTTCCGACCCTGCCGCTTACCGGATACCTGTCCG
CCTTTCTCCCTTCGGGAAGCGTGGCGCTTTCTCATAGCTCACGCTGTAGGTATCTCAGTTCGGTGTAGGTCGTTCGCT
CCAAGCTGGGCTGTGTGCACGAACCCCCCGTTCAGCCCGACCGCTGCGCCTTATCCGGTAACTATCGTCTTGAGTCCA
ACCCGGTAAGACACGACTTATCGCCACTGGCAGCAGCCACTGGTAACAGGATTAGCAGAGCGAGGTATGTAGGCGGTG
CTACAGAGTTCTTGAAGTGGTGGCCTAACTACGGCTACACTAGAAGGACAGTATTTGGTATCTGCGCTCTGCTGAAGC
CAGTTACCTTCGGAAAAAGAGTTGGTAGCTCTTGATCCGGCAAACAAACCACCGCTGGTAGCGGTGGTTTTTTTGTTT
GCAAGCAGCAGATTACGCGCAGAAAAAAAGGATCTCAAGAAGATCCTTTGATCTTTTCTACGGGGTCTGACGCTCAGT
GGAACGAAAACTCACGTTAAGGGATTTTGGTCATGAGATTATCAAAAAGGATCTTCACCTAGATCCTTTTAAATTAAA
AATGAAGTTTTAAATCAATCTAAAGTATATATGAGTAAACTTGGTCTGACAGTTACCAATGCTTAATCAGTGAGGCAC
CTATCTCAGCGATCTGTCTATTTCGTTCATCCATAGTTGCCTGACTCCCCGTCGTGTAGATAACTACGATACGGGAGG
GCTTACCATCTGGCCCCAGTGCTGCAATGATACCGCGAGACCCACGCTCACCGGCTCCAGATTTATCAGCAATAAACC
AGCCAGCCGGAAGGGCCGAGCGCAGAAGTGGTCCTGCAACTTTATCCGCCTCCATCCAGTCTATTAATTGTTGCCGGG
AAGCTAGAGTAAGTAGTTCGCCAGTTAATAGTTTGCGCAACGTTGTTGCCATTGCtgcaGGCATCGTGGTGTCACGCT
CGTCGTTTGGTATGGCTTCATTCAGCTCCGGTTCCCAACGATCAAGGCGAGTTACATGATCCCCCATGTTGTGCAAAA
AAGCGGTTAGCTCCTTCGGTCCTCCGATCGTTGTCAGAAGTAAGTTGGCCGCAGTGTTATCACTCATGGTTATGGCAG
CACTGCATAATTCTCTTACTGTCATGCCATCCGTAAGATGCTTTTCTGTGACTGGTGAGTACTCAACCAAGTCATTCT
GAGAATAGTGTATGCGGCGACCGAGTTGCTCTTGCCCGGCGTCAACACGGGATAATACCGCGCCACATAGCAGAACTT
TAAAAGTGCTCATCATTGGAAAACGTTCTTCGGGGCGAAAACTCTCAAGGATCTTACCGCTGTTGAGATCCAGTTCGA
TGTAACCCACTCGTGCACCCAACTGATCTTCAGCATCTTTTACTTTCACCAGCGTTTCTGGGTGAGCAAAAACAGGAA
GGCAAAATGCCGCAAAAAAGGGAATAAGGGCGACACGGAAATGTTGAATACTCATACTCTTCCTTTTTCAATATTATT
GAAGCATTTATCAGGGTTATTGTCTCATGAGCGGATACATATTTGAATGTATTTAGAAAAATAAACAAATAGGGGTTC
CGCGCACATTTCCCCGAAAAGTGCCACCTGACGTCTAAGAAACCATTATTATCATGACATTAACCTATAAAAATAGGC
GTATCACGAGGCCCTTTCGTCTCGCGCGTTTCGGTGATGACGGTGAAAACCTCTGACACATGCAGCTCCCGGAGACGG
TCACAGCTTGTCTGTAAGCGGATGCCGGGAGCAGACAAGCCCGTCAGGGCGCGTCAGCGGGTGTTGGCGGGTGTCGGG
GCTGGCTTAACTATGCGGCATCAGAGCAGATTGTACTGAGAGTGCACCATatATGCGGTGTGAAATACCGCACAGATG
CGTAAGGAGAAAATACCGCATCAGGCGAAATTGTAAACGTTAATATTTTGTTAAAATTCGCGTTAAATATTTGTTAAA
TCAGCTCATTTTTTAACCAATAGGCCGAAATCGGCAAAATCCCTTATAAATCAAAAGAATAGACCGAGATAGGGTTGA
GTGTTGTTCCAGTTTGGAACAAGAGTCCACTATTAAAGAACGTGGACTCCAACGTCAAAGGGCGAAAAACCGTCTATC
AGGGCGATGGCCCACTACGTGAACCATCACCCAAATCAAGTTTTTTGCGGTCGAGGTGCCGTAAAGCTCTAAATCGGA
ACCCTAAAGGGAGCCCCCGATTTAGAGCTTGACGGGGAAAGCCGGCGAACGTGGCGAGAAAGGAAGGGAAGAAAGCGA
AAGGAGCGGGCGCTAGGGCGCTGGCAAGTGTAGCGGTCACGCTGCGCGTAACCACCACACCCGCCGCGCTTAATGCGC
CGCTACAGGGCGCGTCCATTCGCCATTCAGGCTGCGCAACTGTTGGGAAGGGCGATCGGTGCGGGCCTCTTCGCTATT
ACGCCAGCTGGCGAAAGGGGGATGTGCTGCAAGGCGATTAAGTTGGGTAACGCCAGGGTTTTCCCAGTCACGACGTTG
TAAAACGACGGCCAGTGAATTGTAATACGACTCACTATAGGGC

pFEM136 φC31.Integrase(Ext^{N})-DnaE *Npu* ORF sequence

ATGGATACCTATGCGGGCGCGTATGATCGCCAGAGCCGCGAACGCGAAAACAGCAGCGCGGCGAGCCCGGCGACCCAG
CGCAGCGCGAACGAAGATAAAGCGGCGGATCTGCAGCGCGAAGTGGAACGCGATGGCGGCCGCTTTCGCTTTGTGGGC
CATTTTAGCGAAGCGCCGGGCACCAGCGCGTTTGGCACCGCGGAACGCCCGGAATTTGAACGCATTCTGAACGAATGC
CGCGCGGGCCGCCTGAACATGATTATTGTGTATGATGTGAGCCGCTTTAGCCGCCTGAAAGTGATGGATGCGATTCCG
ATTGTGAGCGAACTGCTGGCGCTGGGCGTGACCATTGTGAGCACCCAGGAAGGCGTGTTTCGCCAGGGCAACGTGATG
GATCTGATTCATCTGATTATGCGCCTGGATGCGAGCCATAAAGAAAGCAGCCTGAAAAGCGCGAAAATTCTGGATACC
AAAAACCTGCAGCGCGAACTGGGCGGCTATGTGGGCGGCAAAGCGCCGTATGGCTTTGAACTGGTGAGCGAAACCAAA
GAAATTACCCGCAACGGCCGCATGGTGAACGTGGTGATTAACAAACTGGCGCATAGCACCACCCCGCTGACCGGCCCG
TTTGAATTTGAACCGGATGTGATTCGCTGGTGGTGGCGCGAAATTAAAACCCATAAACATCTGCCGTTTAAACCGGGC
AGCCAGGCGGCGATTCATCCGGGCAGCATTACCGGCCTGTGCAAACGCATGGATGCGGATGCGGTGCCGACCCGCGGC
GAAACCATTGGCAAAAAAACCGCGAGCAGCGCGTGGGATCCGGCGACCGTGATGCGCATTCTGCGCGATCCGCGCATT
GCGGGCTTTGCGGCGGAAGTGATTTATAAAAAAAAACCGGATGGCACCCCGACCACCAAAATTGAATATTGCCTGAGC
TATGAAACCGAAATTCTGACCGTGGAATATGGCAGCCTGCCGATTGGCAAAATTGTGGAAAAACGCATTGAATGCACC
GTGTATAGCGTGGATAACAACGGCAACATTTATACCCAGCCGGTGGCGCAGTGGCATGATCGCGGCGAACAGGAAGTG
TTTGAATATTGCCTGGAAGATGGCAGCCTGATTCGCGCGACCAAAGATCATAAATTTATGACCGTGGATGGCCAGATG
CTGCCGATTGATGAAATTTTTGAACGCGAACTGGATCTGATGCGCGTGGATAACCTGCCGAACACCAGCCATCATCAT
CATCATCATTAATAA

pFEM155 φC31.Integrase(Ext^{N}) ORF sequence

ATGGATACCTATGCGGGCGCGTATGATCGCCAGAGCCGCGAACGCGAAAACAGCAGCGCGGCGAGCCCGGCGACCCAG
CGCAGCGCGAACGAAGATAAAGCGGCGGATCTGCAGCGCGAAGTGGAACGCGATGGCGGCCGCTTTCGCTTTGTGGGC
CATTTTAGCGAAGCGCCGGGCACCAGCGCGTTTGGCACCGCGGAACGCCCGGAATTTGAACGCATTCTGAACGAATGC
CGCGCGGGCCGCCTGAACATGATTATTGTGTATGATGTGAGCCGCTTTAGCCGCCTGAAAGTGATGGATGCGATTCCG
ATTGTGAGCGAACTGCTGGCGCTGGGCGTGACCATTGTGAGCACCCAGGAAGGCGTGTTTCGCCAGGGCAACGTGATG

GATCTGATTCATCTGATTATGCGCCTGGATGCGAGCCATAAAGAAAGCAGCCTGAAAAGCGCGAAAATTCTGGATACC
AAAAACCTGCAGCGCGAACTGGGCGGCTATGTGGGCGGCAAAGCGCCGTATGGCTTTGAACTGGTGAGCGAAACCAAA
GAAATTACCCGCAACGGCCGCATGGTGAACGTGGTGATTAACAAACTGGCGCATAGCACCACCCCGCTGACCGGCCCG
TTTGAATTTGAACCGGATGTGATTCGCTGGTGGTGGCGCGAAATTAAAACCCATAAACATCTGCCGTTTAAACCGGGC
AGCCAGGCGGCGATTCATCCGGGCAGCATTACCGGCCTGTGCAAACGCATGGATGCGGATGCGGTGCCGACCCGCGGC
GAAACCATTGGCAAAAAAACCGCGAGCAGCGCGTGGGATCCGGCGACCGTGATGCGCATTCTGCGCGATCCGCGCATT
GCGGGCTTTGCGGCGGAAGTGATTTATAAAAAAAAACCGGATGGCACCCCGACCACCAAAATTGAAGGCTATACTAGT
TAATAA

pFEM161 φC31.Integrase(Ext^{N})-DnaE *Npu** ORF sequence

ATGGATACCTATGCGGGCGCGTATGATCGCCAGAGCCGCGAACGCGAAAACAGCAGCGCGGCGAGCCCGGCGACCCAG
CGCAGCGCGAACGAAGATAAAGCGGCGGATCTGCAGCGCGAAGTGGAACGCGATGGCGGCCGCTTTCGCTTTGTGGGC
CATTTTAGCGAAGCGCCGGGCACCAGCGCGTTTGGCACCGCGGAACGCCCGGAATTTGAACGCATTCTGAACGAATGC
CGCGCGGGCCGCCTGAACATGATTATTGTGTATGATGTGAGCCGCTTTAGCCGCCTGAAAGTGATGGATGCGATTCCG
ATTGTGAGCGAACTGCTGGCGCTGGGCGTGACCATTGTGAGCACCCAGGAAGGCGTGTTTCGCCAGGGCAACGTGATG
GATCTGATTCATCTGATTATGCGCCTGGATGCGAGCCATAAAGAAAGCAGCCTGAAAAGCGCGAAAATTCTGGATACC
AAAAACCTGCAGCGCGAACTGGGCGGCTATGTGGGCGGCAAAGCGCCGTATGGCTTTGAACTGGTGAGCGAAACCAAA
GAAATTACCCGCAACGGCCGCATGGTGAACGTGGTGATTAACAAACTGGCGCATAGCACCACCCCGCTGACCGGCCCG
TTTGAATTTGAACCGGATGTGATTCGCTGGTGGTGGCGCGAAATTAAAACCCATAAACATCTGCCGTTTAAACCGGGC
AGCCAGGCGGCGATTCATCCGGGCAGCATTACCGGCCTGTGCAAACGCATGGATGCGGATGCGGTGCCGACCCGCGGC
GAAACCATTGGCAAAAAAACCGCGAGCAGCGCGTGGGATCCGGCGACCGTGATGCGCATTCTGCGCGATCCGCGCATT
GCGGGCTTTGCGGCGGAAGTGATTTATAAGAAGAAGCCGGATGGCACCCCGACCACCAAAATTGGAGCAGCACTGAGC
TATGAAACCGAAATTCTGACCGTGGAATATGGCAGCCTGCCGATTGGCAAAATTGTGGAAAAACGCATTGAATGCACC
GTGTATAGCGTGGATAACAACGGCAACATTTATACCCAGCCGGTGGCGCAGTGGCATGATCGCGGCGAACAGGAAGTG
TTTGAATATTGCCTGGAAGATGGCAGCCTGATTCGCGCGACCAAAGATCATAAATTTATGACCGTGGATGGCCAGATG
CTGCCGATTGATGAAATTTTTGAACGCGAACTGGATCTGATGCGCGTGGATAACCTGCCGAACACCAGCCATCACCAT
CACCATCACTAATAA

pFEM33 φC31.Integrase-gp3 fusion expression plasmid DNA sequence

(The ORF is cloned in the same vector as pFEM141 between NdeI and XhoI sites)

ATGGACACGTACGCGGGTGCTTACGACCGTCAGTCGCGCGAGCGCGAGAATTCGAGCGCAGCAAGCCCAGCGACACAG
CGTAGCGCCAACGAAGACAAGGCGGCCGACCTTCAGCGCGAAGTCGAGCGCGACGGGGGCCGGTTCAGGTTCGTCGGG
CATTTCAGCGAAGCGCCGGGCACGTCGGCGTTCGGGACGGCGGAGCGCCCGGAGTTCGAACGCATCCTGAACGAATGC
CGCGCCGGGCGGCTCAACATGATCATTGTCTATGACGTGTCGCGCTTCTCGCGCCTGAAGGTCATGGACGCGATTCCG
ATTGTCTCGGAATTGCTCGCCCTGGGCGTGACGATTGTTTCCACTCAGGAAGGCGTCTTCCGGCAGGGAAACGTCATG
GACCTGATTCACCTGATTATGCGGCTCGACGCGTCGCACAAAGAATCTTCGCTGAAGTCGGCGAAGATTCTCGACACG
AAGAACCTTCAGCGCGAATTGGGCGGGTACGTCGGCGGGAAGGCGCCTTACGGCTTCGAGCTTGTTTCGGAGACGAAG
GAGATCACGCGCAACGGCCGAATGGTCAATGTCGTCATCAACAAGCTTGCGCACTCGACCACTCCCCTTACCGGACCC
TTCGAGTTCGAGCCCGACGTAATCCGGTGGTGGTGGCGTGAGATCAAGACGCACAAACACCTTCCCTTCAAGCCGGGC
AGTCAAGCCGCCATTCACCCGGGCAGCATCACGGGGCTTTGTAAGCGCATGGACGCTGACGCCGTGCCGACCCGGGGC
GAGACGATTGGGAAGAAGACCGCTTCAAGCGCCTGGGACCCGGCAACCGTTATGCGAATCCTTCGGGACCCGCGTATT
GCGGGCTTCGCCGCTGAGGTGATCTACAAGAAGAAGCCGGACGGCACGCCGACCACGAAGATTGAGGGTTACCGCATT
CAGCGCGACCCGATCACGCTCCGGCCGGTCGAGCTTGATTGCGGACCGATCATCGAGCCCGCTGAGTGGTATGAGCTT
CAGGCGTGGTTGGACGGCAGGGGGCGCGGCAAGGGGCTTTCCCGGGGGCAAGCCATTCTGTCCGCCATGGACAAGCTG
TACTGCGAGTGTGGCGCCGTCATGACTTCGAAGCGCGGGGAAGAATCGATCAAGGACTCTTACCGCTGCCGTCGCCGG
AAGGTGGTCGACCCGTCCGCACCTGGGCAGCACGAAGGCACGTGCAACGTCAGCATGGCGGCACTCGACAAGTTCGTT
GCGGAACGCATCTTCAACAAGATCAGGCACGCCGAAGGCGACGAAGAGACGTTGGCGCTTCTGTGGGAAGCCGCCCGA
CGCTTCGGCAAGCTCACTGAGGCGCCTGAGAAGAGCGGCGAACGGGCGAACCTTGTTGCGGAGCGCGCCGACGCCCTG
AACGCCCTTGAAGAGCTGTACGAAGACCGCGCGGCAGGCGCGTACGACGGACCCGTTGGCAGGAAGCACTTCCGGAAG
CAACAGGCAGCGCTGACGCTCCGGCAGCAAGGGGCGGAAGAGCGGCTTGCCGAACTTGAAGCCGCCGAAGCCCCGAAG
CTTCCCCTTGACCAATGGTTCCCCGAAGACGCCGACGCTGACCCGACCGGCCCTAAGTCGTGGTGGGGGCGCGCGTCA
GTAGACGACAAGCGCGTGTTCGTCGGGCTCTTCGTAGACAAGATCGTTGTCACGAAGTCGACTACGGGCAGGGGGCAG
GGAACGCCCATCGAGAAGCGCGCTTCGATCACGTGGGCGAAGCCGCCGACCGACGACGACGAAGACGACGCCCAGGAC
GGCACGGAAGACGTAGCGGCGACTAGTAGATCTGGTACCGCGAAGCGTTCGATCTGGGCCGGAGACGAAGACAACAAG
CCGAAGAAGCGTGAGACGTACGCCGACGACACCGTTGGGCGTTTCCACTCCGGCTACAGCGAGACGAATGAGCGGGGC
AAGGTCGTTCCCGTCGCGCTCGACAAGTGGCGTATCTCGACCGGCGAACAGTCCGTTGCGGACGCTGTGGCTCAGCTC
TTCGGCGGCACCCCGGTTGAGAACGAAGAGAGCACGTCTGAGAACTTCATCGACGTGTTCACCGACCGGCCGAAGGTC
CCCGTGATCATTGAGGCTGACGGTATCCACTGGGATATGAAGCTTTGGCTGAACGGCAAGCTGAAGCACCACTGCGAC
GGCTTTGACTTCGTGTCGCACGCCGACGAAGAGATGATCGGTCAGCCGTGCGGGTGCCCGAAGCTCTTCGATGAGCGG
AAGGCAGCGGCGAAGGAGTACGACGCGCCGAACCCGGCAATCACCGTGACCTTCACGCTCGCCGACGACCCGGAGCTT
GGCCGCTTCAAGTTCCAGACCGGTTCTTGGACGCTCTTCAAGGTTCTGCACGAAGCCGAAGACGACGTTGAGCGCGTC
GGCAAGGGTGGCGCTGTGCTCGCCAATCTCGAACTTGAGCTTGTGGAGTACACGCCGAAGCGTGGCCCGATGCGGAAC
AAGCTTGTGAGCTACTACAAGCCGACCATCACGGTTCTGAAGTCGTACAACGACGCGATTGCCGACTAATAA**CTCGAG**

(b) *In vivo* expression vectors derived from pACYC184

In these plasmids, protein-coding DNA sequences were inserted between NdeI and Acc65I sites in a plasmid derived from pACYC184 carrying a p15a origin of replication (Olorunniji *et al.*, 2017),

pFEM137 DnaE-*Ssp*-φC31.Integrase(Ext^{N}) expression plasmid DNA sequence

GAATTCTAGAAATAATTTTGTTTAACTTTAAGAAGGAGATATA**CATATG**GTGAAAGTGATTGGCCGCCGCAGCCTGGG
CGTGCAGCGCATTTTTGATATTGGCCTGCGCCAGGATCATAACTTTCTGCTGGCGAACGGCGCGATTGCGGCGAACTG
CTTTAACCGCGATCCGATTACCCTGCGCCCGGTGGAACTGGATTGCGGCCCGATTATTGAACCGGCGGAATGGTATGA
ACTGCAGGCGTGGCTGGATGGCCGCGGCCGCGGCAAAGGCCTGAGCCGCGGCCAGGCGATTCTGAGCGCGATGGATAA
ACTGTATTGCGAATGCGGCGCGGTGATGACCAGCAAACGCGGCGAAGAAAGCATTAAAGATAGCTATCGCTGCCGCCG
CCGCAAAGTGGTGGATCCGAGCGCGCCGGGCCAGCATGAAGGCACCTGCAACGTGAGCATGGCGGCGCTGGATAAATT
TGTGGCGGAACGCATTTTTAACAAAATTCGCCATGCGGAAGGCGATGAAGAAACCCTGGCGCTGCTGTGGGAAGCGGC
GCGCCCGCTTTGGCAAACTGACCGAAGCGCCGGAAAAAAGCGGCGAACGCGCGAACCTGGTGGCGGAACGCGCGGATGC
GCTGAACGCGCTGGAAGAACTGTATGAAGATCGCGCGGCGGGCGCGTATGATGGCCCGGTGGGCCGCAAACATTTTCG
CAAACAGCAGGCGGCGCTGACCCTGCGCCAGCAGGGCGCGGAAGAACGCCTGGCGGAACTGGAAGCGGCGGAAGCGCC
GAAACTGCCGCTGGATCAGTGGTTTCCGGAAGATGCGGATGCGGATCCGACCGGCCCGAAAAGCTGGTGGGGCCGCGC
GAGCGTGGATGATAAACGCGTGTTTGTGGGCCTGTTTGTGGATAAAATTGTGGTGACCAAAAGCACCACCGGCCGCGG
CCAGGGCACCCCGATTGAAAAACGCGCGAGCATTACCTGGGCGAAACCGCCGACCGATGATGATGAAGATGATGCGCA
GGATGGCACCGAAGATGTGGCGGCGACCAGCCATCATCATCATCATCATTAATAA**CTCGAG**GGTACCTCTAGAGCTTG
AGTATTCTATAGTGTCACCTAAATAGCTTGGCGTAATCATGGTCATAGCTGTTTCCTGTGTGAAATTGTTATCCGCTC
ACAATTCCACACAACATACGAGCCGGAAGCATAAAGTGTAAAGCCTGGGGTGCCTAATGAGTGAGCTAACTCACATTA
ATTGCGTTGCGCTCACTGCCCGCTTTCCAGTCGGGAAACCTGTCGTGCCAGCTGCATTAATGAATCGGCCAACGCGCG
GGGAGAGGCGGTTTGCGTATTGGGCGCTCTTCCGCTTCCTCGCTCACTGACTCGCTGCGCTCGGTCGTTCGGCTGCGG
CGAGCGGTATCAGCTCACTCAAAGGCGGTAATACGGTTATCCACAGAATCAGGGGATAACGCAGGAAAGAACATGAAT
TATCCCGCAAGAGGCCCGGCAGTACCGGCATAACCAAGCCTATGCCTACAGCATCCAGGGTGACGGTGCCGAGGATGA
CGATGAGCGCATTGTTAGATTTCATACACGGTGCCTGACTGCGTTAGCAATTTAACTGTGATAAACTACCGCATTAAA
GCTTATCGATGATAAGCTGTCAAACATGAGAATTACAACTTATATCGTATGGGGCTGACTTCAGGTGCTACATTTGAA
GAGATAAATTGCACTGAAATCTAGAAATATTTTATCTGATTAATAAGATGATCTTCTTGAGATCGTTTTGGTCTGCGC
GTAATCTCTTGCTCTGAAAACGAAAAAACCGCCTTGCAGGGCGGTTTTTCGAAGGTTCTCTGAGCTACCAACTCTTTG
AACCGAGGTAACTGGCTTGGAGGAGCGCAGTCACCAAAACTTGTCCTTTCAGTTTAGCCTTAACCGGCGCATGACTTC
AAGACTAACTCCTCTAAATCAATTACCAGTGGCTGCTGCCAGTGGTGCTTTTGCATGTCTTTCCGGGTTGGACTCAAG
ACGATAGTTACCGGATAAGGCGCAGCGGTCGGACTGAACGGGGGGTTCGTGCATACAGTCCAGCTTGGAGCGAACTGC
CTACCCGGAACTGAGTGTCAGGCGTGGAATGAGACAAACGCGGCCATAACAGCGGAATGACACCGGTAAACCGAAAGG
CAGGAACAGGAGAGCGCACGAGGGAGCCGCCAGGGGGAAACGCCTGGTATCTTTATAGTCCTGTCGGGTTTCGCCACC
ACTGATTTGAGCGTCAGATTTCGTGATGCTTGTCAGGGGGGCGGAGCCTATGGAAAAACGGCTTTGCCGCGGCCCTCT
CACTTCCCTGTTAAGTATCTTCCTGGCATCTTCCAGGAAATCTCCGCCCCGTTCGTAAGCCATTTCCGCTCGCCGCAG
TCGAACGACCGAGCGTAGCGAGTCAGTGAGCGAGGAAGCGGAATATATCCTGTATCACATATTCTGCTGACGCACCGG
TGCAGCCTTTTTTCTCCTGCCACATGAAGCACTTCACTGACACCCTCATCAGTGCCAACATAGTAAGCCAGTATACAC
TCCGCTAGCGCTGATGTCCGGCGGTGCTTTTGCCGTTACGCACCACCCCGTCAGTAGCTGAACAGGAGGGACAGCTGA
TAGAAACAGAAGCCACTGGAGCACCTCAAAAACACCATCATACACTAAATCAGTAAGTTGGCAGCATCACCCGACGCA
CTTTGCGCCGAATAAATACCTGTGACGGAAGATCACTTCGCAGAATAAATAAATCCTGGTGTCCCTGTTGATACCGGG
AAGCCCTGGGCCAACTTTTGGCGAAAATGAGACGTTGATCGGCACGTAAGAGGTTCCAACTTTCACCATAATGAAATA
AGATCACTACCGGGCGTATTTTTTGAGTTATCGAGATTTTCAGGAGCTAAGGAAGCTAAAATGGAGAAAAAAATCACT
GGATATACCACCGTTGATATATCCCAATGGCATCGTAAAGAACATTTTGAGGCATTTCAGTCAGTTGCTCAATGTACC
TATAACCAGACCGTTCAGCTGGATATTACGGCCTTTTTAAAGACCGTAAAGAAAAATAAGCACAAGTTTTATCCGGCC
TTTATTCACATTCTTGCCCGCCTGATGAATGCTCATCCGGAATTCCGTATGGCAATGAAAGACGGTGAGCTGGTGATA
TGGGATAGTGTTCACCCTTGTTACACCGTTTTCCATGAGCAAACTGAAACGTTTTCATCGCTCTGGAGTGAATACCAC
GACGATTTCCGGCAGTTTCTACACATATATTCGCAAGATGTGGCGTGTTACGGTGAAAACCTGGCCTATTTCCCTAAA
GGGTTTATTGAGAATATGTTTTTCGTCTCAGCCAATCCCTGGGTGAGTTTCACCAGTTTTGATTTAAACGTGGCCAAT
ATGGACAACTTCTTCGCCCCCGTTTTCACCATGGGCAAATATTATACGCAAGGCGACAAGGTGCTGATGCCGCTGGCG
ATTCAGGTTCATCATGCCGTCTGTGATGGCTTCCATGTCGGCAGAATGCTTAATGAATTACAACAGTACTGCGATGAG
TGGCAGGGCGGGGCGTAATTTTTTTAAGGCAGTTATTGGTGCCCTTAAACGCCTGGTGCTACGCCTGAATAAGTGATA
ATAAGCGGATGAATGGCAGAAATTCGAAAGCAAATTCGACCCGGTCGTCGGTTCAGGGCAGGGTCGTTAAATAGCCGC
TTATGTCTATTGCTGGTTTACCGGTTTATTGACTACCGGAAGCAGTGTGACCGTGTGCTTCTCAAATGCCTGAGGCCA
GTTTGCTCAGGCTCTCCCCGTGGAGGTAATAATTGACGATATGATCATTTATTCTGCCTCCCAGAGCCTGATAAAAAC
GGTTAGCGCTTCGTTAATACAGATGTAGGTGTTCCACAGGGTAGCCAGCAGCATCCTGCGATGCAGATCCGGAACATA
ATGGTGCAGGGCGCTTGTTTCGGCGTGGGTATGGTGGCAGGCCCCGTGGCCGGGGGACTGTTGGGCGCTGCCGGCACC
TGTCCTACGAGTTGCATGATAAAGAAGACAGTCATAAGTGCGGCGACGATAGTCATGCCCCGCGCCCACCGGAAGGAG
CTACCGGACAGCGGTGCGGACTGTTGTAACTCAGAATAAGAAATGAGGCCGCTCATGGCGTTGACTCTCAGTCATAGT
ATCGTGGTATCACCGGTTGGTTCCACTCTCTGTTGCGGGCAACTTCAGCAGCACGTAGGGGACTTCCGCGTTTCCAGA
CTTTACGAAACACGGAAACCGAAGACCATTCATGTTGTTGCTCAGGTCGCAGACGTTTTGCAGCAGCAGTCGCTTCAC

GTTCGCTCGCGTATCGGTGATTCATTCTGCTAACCAGTAAGGCAACCCCGCCAGCCTAGCCGGGTCCTCAACGACAGG
AGCACGATCATGCGCACCCGTGGCCAGGACCCAACGCTGCCCGAGATGCGCCGCGTGCGGCTGCTGGAGATGGCGGAC
GCGATGGATATGTTCTGCCAAGGGTTGGTTTGCGCATTCACAGTTCTCCGCAAGAATTGATTGGCTCCAATTCTTGGA
GTGGTGAATCCGTTAGCGAGGTGCCGCCGGCTTCCATTCAGGTCGAGGTGGCCCGGCTCCATGCACCGCGACGCAACG
CGGGGAGGCAGACAAGGTATAGGGCGGCGCCTACAATCCATGCCAACCCGTTCCATGTGCTCGCCGAGGCGGCATAAA
TCGCCGTGACGATCAGCGGTCCAGTGATCGAAGTTAGGCTGGTAAGAGCCGCGAGCGATCCTTGAAGCTGTCCCTGAT
GGTCGTCATCTACCTGCCTGGACAGCATGGCCTGCAACGCGGGCATCCCGATGCCGCCGGAAGCGAGAAGAATCATAA
TGGGGAAGGCCATCCAGCCTCGCGTCGCGAACGCCAGCAAGACGTAGCCCAGCGCGTCGGCCGCCATGCCGGCGATAA
TGGCCTGCTTCTCGCCGAAACGTTTGGTGGCGGGACCAGTGACGAAGGCTTGAGCGAGGGCGTGCAAGATTCCGAATA
CCGCAAGCGACAGGCCGATCATCGTCGCGCTCCAGCGAAAGCGGTCCTCGCCGAAAATGACCCAGAGCGCTGCCGGCA
CCTGTCCTACGAGTTGCATGATAAAGAAGACAGTCATAAGTGCGGCGACGATAGTCATGCCCCGCGCCCACCGGAAGG
AGCTGACTGGGTTGAAGGCTCTCAAGGGCATCGGTCgagAACGACGGCCAGTGAATagtcatACGACTCACTATAGGG
C

pFEM157 φC31.Integrase(Ext^C) expression plasmid DNA sequence

ATGCGTATCCAGCGCGATCCGATTACCCTGCGCCCGGTGGAACTGGATTGCGGCCCGATTATTGAACCGGCGGAATGG
TATGAACTGCAGGCGTGGCTGGATGGCCGCGGCCGCGGCAAAGGCCTGAGCCGCGGCCAGGCGATTCTGAGCGCGATG
GATAAACTGTATTGCGAATGCGGCGCGGTGATGACCAGCAAACGCGGCGAAGAAAGCATTAAAGATAGCTATCGCTGC
CGCCGCCGCAAAGTGGTGGATCCGAGCGCGCCGGGCCAGCATGAAGGCACCTGCAACGTGAGCATGGCGGCGCTGGAT
AAATTTGTGGCGGAACGCATTTTTAACAAAATTCGCCATGCGGAAGGCGATGAAGAAACCCTGGCGCTGCTGTGGGAA
GCGGCGCGCCGCTTTGGCAAACTGACCGAAGCGCCGGAAAAAAGCGGCGAACGCGCGAACCTGGTGGCGGAACGCGCG
GATGCGCTGAACGCGCTGGAAGAACTGTATGAAGATCGCGCGGCGGGCGCGTATGATGGCCCGGTGGGCCGCAAACAT
TTTCGCAAACAGCAGGCGGCGCTGACCCTGCGCCAGCAGGGCGCGGAAGAACGCCTGGCGGAACTGGAAGCGGCGGAA
GCGCCGAAACTGCCGCTGGATCAGTGGTTTCCGGAAGATGCGGATGCGGATCCGACCGGCCCGAAAAGCTGGTGGGGC
CGCGCGAGCGTGGATGATAAACGCGTGTTTGTGGGCCTGTTTGTGGATAAAATTGTGGTGACCAAAAGCACCACCGGC
CGCGGCCAGGGCACCCCGATTGAAAAACGCGCGAGCATTACCTGGGCGAAACCGCCGACCGATGATGATGAAGATGAT
GCGCAGGATGGCACCGAAGATGTGGCGGCGACCAGCCATCATCATCATCATCATTAATAA

pFEM162 DnaE-*Ssp**-φC31.Integrase(Ext^N) expression plasmid DNA sequence

ATGGTGAAAGTGATTGGCCGCCGCAGCCTGGGCGTGCAGCGCATTTTTGATATTGGCCTGCGCCAGGATCATAACTTT
CTGCTGGCGAACGGCGCGATTGCGGCGAACGCATCAGCACGCGATCCGATTACCCTGCGCCCGGTGGAACTGGATTGC
GGCCCGATTATTGAACCGGCGGAATGGTATGAACTGCAGGCGTGGCTGGATGGCCGCGGCCGCGGCAAAGGCCTGAGC
CGCGGCCAGGCGATTCTGAGCGCGATGGATAAACTGTATTGCGAATGCGGCGCGGTGATGACCAGCAAACGCGGCGAA
GAAAGCATTAAAGATAGCTATCGCTGCCGCCGCCGCAAAGTGGTGGATCCGAGCGCGCCGGGCCAGCATGAAGGCACC
TGCAACGTGAGCATGGCGGCGCTGGATAAATTTGTGGCGGAACGCATTTTTAACAAAATTCGCCATGCGGAAGGCGAT
GAAGAAACCCTGGCGCTGCTGTGGGAAGCGGCGCGCCGCTTTGGCAAACTGACCGAAGCGCCGGAAAAAAGCGGCGAA
CGCGCGAACCTGGTGGCGGAACGCGCGGATGCGCTGAACGCGCTGGAAGAACTGTATGAAGATCGCGCGGCGGGCGCG
TATGATGGCCCGGTGGGCCGCAAACATTTTCGCAAACAGCAGGCGGCGCTGACCCTGCGCCAGCAGGGCGCGGAAGAA
CGCCTGGCGGAACTGGAAGCGGCGGAAGCGCCGAAACTGCCGCTGGATCAGTGGTTTCCGGAAGATGCGGATGCGGAT
CCGACCGGCCCGAAAAGCTGGTGGGGCCGCGCGAGCGTGGATGATAAACGCGTGTTTGTGGGCCTGTTTGTGGATAAA
ATTGTGGTGACCAAAAGCACCACCGGCCGCGGCCAGGGCACCCCGATTGAAAAACGCGCGAGCATTACCTGGGCGAAA
CCGCCGACCGATGATGATGAAGATGATGCGCAGGATGGCACCGAAGATGTGGCGGCGACCAGCCATCATCATCATCAT
CATTAATAA

pFEM188 DnaE-*Ssp*-φC31.Integrase(Ext^N)-gp3 expression plasmid DNA sequence

ATGGTGAAAGTGATTGGCCGCCGCAGCCTGGGCGTGCAGCGCATTTTTGATATTGGCCTGCGCCAGGATCATAACTTT
CTGCTGGCGAACGGCGCGATTGCGGCGAACTGCTTTAACCGCGATCCGATTACCCTGCGCCCGGTGGAACTGGATTGC
GGCCCGATTATTGAACCGGCGGAATGGTATGAACTGCAGGCGTGGCTGGATGGCCGCGGCCGCGGCAAAGGCCTGAGC
CGCGGCCAGGCGATTCTGAGCGCGATGGATAAACTGTATTGCGAATGCGGCGCGGTGATGACCAGCAAACGCGGCGAA
GAAAGCATTAAAGATAGCTATCGCTGCCGCCGCCGCAAAGTGGTGGATCCGAGCGCGCCGGGCCAGCATGAAGGCACC
TGCAACGTGAGCATGGCGGCGCTGGATAAATTTGTGGCGGAACGCATTTTTAACAAAATTCGCCATGCGGAAGGCGAT
GAAGAAACCCTGGCGCTGCTGTGGGAAGCGGCGCGCCGCTTTGGCAAACTGACCGAAGCGCCGGAAAAAAGCGGCGAA
CGCGCGAACCTGGTGGCGGAACGCGCGGATGCGCTGAACGCGCTGGAAGAACTGTATGAAGATCGCGCGGCGGGCGCG
TATGATGGCCCGGTGGGCCGCAAACATTTTCGCAAACAGCAGGCGGCGCTGACCCTGCGCCAGCAGGGCGCGGAAGAA
CGCCTGGCGGAACTGGAAGCGGCGGAAGCGCCGAAACTGCCGCTGGATCAGTGGTTTCCGGAAGATGCGGATGCGGAT
CCGACCGGCCCGAAAAGCTGGTGGGGCCGCGCGAGCGTGGATGATAAACGCGTGTTTGTGGGCCTGTTTGTGGATAAA
ATTGTGGTGACCAAAAGCACCACCGGCCGCGGCCAGGGCACCCCGATTGAAAAACGCGCGAGCATTACCTGGGCGAAA
CCGCCGACCGATGATGATGAAGATGATGCGCAGGATGGCACCGAAGATGTGGCGGCGACTAGTAGATCTGGCACCGCG
AAGCGTTCGATCTGGGCCGGAGACGAAGACAACAAGCCGAAGAAGCGTGAGACGTACGCCGACGACACCGTTGGGCGT
TTCCACTCCGGCTACAGCGAGACGAATGAGCGGGGCAAGGTCGTTCCCGTCGCGCTCGACAAGTGGCGTATCTCGACC
GGCGAACAGTCCGTTGCGGACGCTGTGGCTCAGCTCTTCGGCGGCACCCCGGTTGAGAACGAAGAGAGCACGTCTGAG
AACTTCATCGACGTGTTCACCGACCGGCCGAAGGTCCCCGTGATCATTGAGGCTGACGGTATCCACTGGGATATGAAG
CTTTGGCTGAACGGCAAGCTGAAGCACCACTGCGACGGCTTTGACTTCGTGTCGCACGCCGACGAAGAGATGATCGGT

CAGCCGTGCGGGTGCCCGAAGCTCTTCGATGAGCGGAAGGCAGCGGCGAAGGAGTACGACGCGCCGAACCCGGCAATC
ACCGTGACCTTCACGCTCGCCGACGACCCGGAGCTTGGCCGCTTCAAGTTCCAGACCGGTTCTTGGACGCTCTTCAAG
GTTCTGCACGAAGCCGAAGACGACGTTGAGCGCGTCGGCAAGGGTGGCGCTGTGCTCGCCAATCTCGAACTTGAGCTT
GTGGAGTACACGCCGAAGCGTGGCCCGATGCGGAACAAGCTTGTGAGCTACTACAAGCCGACCATCACGGTTCTGAAG
TCGTACAACGACGCGATTGCCGACACCAGCCATCATCATCATCATCATTAATAA

(e) *In vivo* recombination substrate plasmids

pFM211 φC31 integrase attP x attB inversion substrate

(attP, yellow; attB, cyan; RFP, red; GFP, green; J23104 promoter, pink)

TGGCCGGCGTAAGTGGATTTACCATAATCCCTTAATTGTACGCACCGCTAAAACGCGTTCAGCGCGATCACGGCAGCA
GACAGGTAAAAATGGCAACAAACCACCCGAAAAACTGCCGCGATCGCGCCTGATAAATTTTAACCGTATGAATACCTA
TGCAACCAGAGGGTACAGGCCACATTACCCCCACTTAATCCACTGAAGCTGCCATTTTTCATGGTTTCACCATCCCAG
CGAAATTCGAACTGAAGCTAAGAGCGCAGTATAGTAGTTTCAAATAAAGCTGAGACGCTAAATCGTGAGACCAAATAA
TAAAAAAGCCGGATTAATAATCTGGCTTTTTATATTCTCTGGTACCTTATTACTTATAGAGCTCGTCCATTCCGTGCG
TAATGCCAGCTGCGGTCACAAACTCCAAGAGCACCATGTGATCGCGTTTCTCATTGGGGTCTTTCGACAAGACGCTTT
GGGTGGACAGATAATGGTTATCCGGCAGTAAGACAGGGCCATCACCAATCGGTGTATTCTGCTGGTAATGATCGGCCA
GTTGAACTGACCCATCTTCCACGTTATGGCGAATTTTGAAGTTCGCTTTGATGCCATTCTTCTGTTTATCGGCAGTGA
TGTAAACGTTATGCGAGTTAAAGTTGTACTCCAGTTTATGCCCCAGAATATTGCCATCTTCCTTAAAGTCGATGCCTT
TCAGTTCGATGCGATTGACCAGGGTGTCACCTTCAAATTTCACCTCTGCGCGGGTTTTGTAGGTCCCATCGTCTTTAA
AGCTGATGGTCCGTTCTTGCACATAGCCTTCCGGCATCGCAGATTTGAAGAAATCGTGCTGTTTCATGTGGTCTGGAT
AACGAGCAAAGCACTGCACACCATACGTAAGGGTAGTCACAAGAGTTGGCCACGGTACAGGTAACTTGCCTGTCGTAC
AAATGAACTTGAGGGTCAGTTTACCATTCGTCGCGTCACCTTCACCTTCACCACGAACACTGAATTTATGTCCGTTGA
CATCGCCATCCAGTTCAACCAGGATCGGTACTACACCCGTAAACAGTTCCTCGCCTTTACGCATATGGTATTTCTCCT
CTTTCTCTAGTGCTAGCCCGCGTACGCCCCCAACTGAGAGAACTCAAAGGTTACCCCAGTTGGGGCACTACTCTCGAG
GCCGCGTTGGCGAGTCGGCTAAGCCGTGTGAATGGATATCTCTAGGGCGGCGGATTTGTCCTACTCAGGAGAGCGTTC
ACCGACAAACAACAGATAAAACGAAAGGCCCAGTCTTTCGACTGAGCCTTTCGTTTTATTTGATGCCGCGGCCGCCTC
ATCATCCCTATCACGGTTTCGAGTGACTCGACCGAACGCGAGCATCGCAAATTGTGTCCGCTGAGACAACTCCGTAGT
TGACTACGCATCCCTCTAGGCCTTACTTAACCGGATACAGTGACTTTGACAGGTTTGTGGGCTACAGCAATCACTTGC
ATAGCTGCGTATGGTACAGGAAGCAACTCTTCTAGATTGACAGCTAGATCAGTCCTAGGTATTGTGCCAGCGGTAACC
CCGCGGTGCGGGTGCCAGGGCGTGCCCTTGGGCTCCCCGGGCGCGTACTCCACCTGGGCCCACACATACTAGAGAAAG
AGGAGAAATACTAGATGGCTTCCTCCGAAGACGTTATCAAAGAGTTCATGCGTTTCAAAGTTCGTATGGAAGGTTCCG
TTAACGGTCACGAGTTCGAAATCGAAGGTGAAGGTGAAGGTCGTCCGTACGAAGGTACCCAGACCGCTAAACTGAAAG
TTACCAAAGGTGGTCCGCTGCCGTTCGCTTGGGACATCCTGTCCCCGCAGTTCCAGTACGGTTCCAAAGCTTACGTTA
AACACCCGGCTGACATCCCGGACTACCTGAAACTGTCCTTCCCGGAAGGTTTCAAATGGGAACGTGTTATGAACTTCG
AAGACGGTGGTGTTGTTACCGTTACCCAGGACTCCTCCCTGCAAGACGGTGAGTTCATCTACAAAGTTAAACTGCGTG
GTACCAACTTCCCGTCCGACGGTCCGGTTATGCAGAAAAAAACCATGGGTTGGGAAGCTTCCACCGAACGTATGTACC
CGGAAGACGGTGCTCTGAAAGGTGAAATCAAAATGCGTCTGAAACTGAAAGACGGTGGTCACTACGACGCTGAAGTTA
AAACCACCTACATGGCTAAAAAACCGGTTCAGCTGCCGGGTGCTTACAAAACCGACATCAAACTGGACATCACCTCCC
ACAACGAAGACTACACCATCGTTGAACAGTACGAACGTGCTGAAGGTCGTCACTCCACCGGTGCTTAATAATGTACAT
TTCTTTTGGGTATAGCGTCGTGGACAGTCATTCATCTTTCTGCCCCTCCAAAAGCAAAAACCCGCCGAAGCGGGTTTT
TACGTAAATCAGGTGAAACTGACCGATAAGCCGGGACGTCACCGGTAAATTCTCTCATCGTTAATTGAAATGACTATA
CTGCGCTCTTAGCTTCAGCCATGGCGGTGAACAGTTGTTCTACTTTTGTTTGTTAGTCTTGATGCTTCACTGATAGAT
ACAAGAGCCATAAGAACCTCAGATCCTTCCGTATTTAGCCAGTATGTTCTCTAGTGTGGTTCGTTGTTTTTGCGTGAG
CCATGAGAACGAACCATTGAGATCATGCTTACTTTGCATGTCACTCAAAAATTTTGCCTCAAAACTGGTGAGCTGAAT
TTTTGCAGTTAAAGCATCGTGTAGTGTTTTTCTTAGTCCGTTACGTAGGTAGGAATCTGATGTAATGGTTGTTGGTAT
TTTGTCACCATTCATTTTTATCTGGTTGTTCTCAAGTTCGGTTACGAGATCCATTTGTCTATCTAGTTCAACTTGGAA
AATCAACGTATCAGTCGGGCGGCCTCGCTTATCAACCACCAATTTCATATTGCTGTAAGTGTTTAAATCTTTACTTAT
TGGTTTCAAAACCCATTGGTTAAGCCTTTTAAACTCATGGTAGTTATTTTCAAGCATTAACATGAACTTAAATTCATC
AAGGCTAATCTCTATATTTGCCTTGTGAGTTTTCTTTTGTGTTAGTTCTTTTAATAACCACTCATAAATCCTCATAGA
GTATTTGTTTTCAAAAGACTTAACATGTTCCAGATTATATTTTATGAATTTTTTTAACTGGAAAAGATAAGGCAATAT
CTCTTCACTAAAAACTAATTCTAATTTTTCGCTTGAGAACTTGGCATAGTTTGTCCACTGGAAAATCTCAAAGCCTTT
AACCAAAGGATTCCTGATTTCCACAGTTCTCGTCATCAGCTCTCTGGTTGCTTTAGCTAATACACCATAAGCATTTTC
CCTACTGATGTTCATCATCTGAGCGTATTGGTTATAAGTGAACGATACCGTCCGTTCTTTCCTTGTAGGGTTTTCAAT
CGTGGGGTTGAGTAGTGCCACACAGCATAAAATTAGCTTGGTTTCATGCTCCGTTAAGTCATAGCGACTAATCGCTAG
TTCATTTGCTTTGAAAACAACTAATTCAGACATACATCTCAATTGGTCTAGGTGATTTTAATCACTATACCAATTGAG
ATGGGCTAGTCAATGATAATTACTAGTCCTTTTCCTTTGAGTTGTGGGTATCTGTAAATTCTGCTAGACCTTTGCTGG
AAAACTTGTAAATTCTGCTAGACCCTCTGTAAATTCCGCTAGACCTTTGTGTGTTTTTTTTGTTTATATTCAAGTGGT
TATAATTTATAGAATAAAGAAAGAATAAAAAAAGATAAAAAGAATAGATCCCAGCCCTGTGTATAACTCACTACTTTA
GTCAGTTCCGCAGTATTACAAAAGGATGTCGCAAACGCTGTTTGCTCCTCTACAAAACAGACCTTAAAACCCTAAAGG

CTTAAGTAGCACCCTCGCAAGCTCGGGCAAATCGCTGAATATTCCTTTTGTCTCCGACCATCAGGCACCTGAGTCGCT
GTCTTTTTCGTGACATTCAGTTCGCTGCGCTCACGGCTCTGGCAGTGAATGGGGGTAAATGGCACTACAGGCGCCTTT
TATGGATTCATGCAAGGAAACTACCCATAATACAAGAAAAGCCCGTCACGGGCTTCTCAGGGCGTTTTATGGCGGGTC
TGCTATGTGGTGCTATCTGACTTTTTGCTGTTCAGCAGTTCCTGCCCTCTGATTTTCCAGTCTGACCACTTCGGATTA
TCCCGTGACAGGTCATTCAGACTGGCTAATGCACCCAGTAAGGCAGCGGTATCATCAACAGGCTTACCCGTCTTACTG
TCCCGGATCCGTCGACCTGCAGGGGGGGGGGGGGCGCTGAGGTCTGCCTCGTGAAGAAGGTGTTGCTGACTCATACCAG
GCCTGAATCGCCCCATCATCCAGCCAGAAAGTGAGGGAGCCACGGTTGATGAGAGCTTTGTTGTAGGTGGACCAGTTG
GTGATTTTGAACTTTTGCTTTGCCACGGAACGGTCTGCGTTGTCGGGAAGATGCGTGATCTGATCCTTCAACTCAGCA
AAAGTTCGATTTATTCAACAAAGCCGCCGTCCCGTCAAGTCAGCGTAATGCTCTGCCAGTGTTACAACCAATTAACCA
ATTCTGATTAGAAAAACTCATCGAGCATCAAATGAAACTGCAATTTATTCATATCAGGATTATCAATACCATATTTTT
GAAAAAGCCGTTTCTGTAATGAAGGAGAAAACTCACCGAGGCAGTTCCATAGGATGGCAAGATCCTGGTATCGGTCTG
CGATTCCGACTCGTCCAACATCAATACAACCTATTAATTTCCCCTCGTCAAAAATAAGGTTATCAAGTGAGAAATCAC
CATGAGTGACGACTGAATCCGGTGAGAATGGCAAAAGCTTATGCATTTCTTTCCAGACTTGTTCAACAGGCCAGCCAT
TACGCTCGTCATCAAAATCACTCGCATCAACCAAACCGTTATTCATTCGTGATTGCGCCTGAGCGAGACGAAATACGC
GATCGCTGTTAAAAGGACAATTACAAACAGGAATCGAATGCAACCGGCGCAGGAACACTGCCAGCGCATCAACAATAT
TTTCACCTGAATCAGGATATTCTTCTAATACCTGGAATGCTGTTTTCCCGGGGATCGCAGTGGTGAGTAACCATGCAT
CATCAGGAGTACGGATAAAATGCTTGATGGTCGGAAGAGGCATAAATTCCGTCAGCCAGTTTAGTCTGACCATCTCAT
CTGTAACATCATTGGCAACGCTACCTTTGCCATGTTTCAGAAACAACTCTGGCGCATCGGGCTTCCCATACAATCGAT
AGATTGTCGCACCTGATTGCCCGACATTATCGCGAGCCCATTTATACCCATATAAATCAGCATCCATGTTGGAATTTA
ATCGCGGCCTCGAGCAAGACGTTTCCCGTTGAATATGGCTCATAACACCCCTTGTATTACTGTTTATGTAAGCAGACA
GTTTTATTGTTCATGATGATATATTTTTATCTTGTGCAATGTAACATCAGAGATTTTGAGACACAACGTGGCTTTCCC
CCCCCCCCTGCAGGCATCGTGGTGTCACGCTCGGCA

pFM212 φC31 integrase attR x attL inversion substrate

FM212 has identical sequence to pFM211 but with attP and attB sequences replaced with attR and attL respectively and in the same inverted repeat (inversion) orientation.

pFM152 φC31 integrase attP x attB deletion substrate

(attP, yellow; attB, cyan)

GCATCGTGGTGTCACGCTCGGCAtggccggcGCTAGCAAGATCTTCTAGTTACGCCCCCAACTGAGAGAACTCAAAGG
TTACCCCAGTTGGGGCACTACTGCGGCCGCGCATGCATCGATAGATCCAATTGCCGTGACGCAGGCATGTTTCTCAAT
AACGAAATTTGATAAAATCCCGCTCTTTCATAACATTATTTCAGCCTTCTTCAGGGCTGACTGTTTGCATAAAAATTC
ATCTGTATGCACAATAATGTTGTATCAACCACCATATCGGGTGACTTATGCGAAGCTCGGCTAAGCAAGAAGATTAAT
TAATAAAGCATTTGGGCGAACGCGAATTAATTCACTGGCCGTCGTTTTACAACGTCGTGACTGGGAAAACCCTGGCGT
TACCCAACTTAATCGCCTTGCAGCACATCCCCCTTTCGCCAGGGGCAATAAGGGCTGCACGCGCACTTTTATCCGCCT
CTGCTGCGCTCCGCCACCGTACGTAAATTTATGGTTGGTTATGAAATGCTGGCAGAGACCCAGCGAGACCTGACCGCA
GAACAGGCAGCAGAGCGTTTGCGCGCAGTCAGCGATATCCATTTTCGCGAATCCGGAGTGTAAGAAATGAGTCTGAAA
GAAAAAACACAATCTCTGTTTGCCAACGCATTTGGCTACCCTGCCACTCACACCATTCAGGCGCCTGGCCGCGTGAAT
TTGATTGGTGAACACACCGACTACAACGACGGTTTCGTTCTGCCCTGCGCGATTGATTATCAAACCGTGATCAGTTGT
GCACCACGCGATGACCGTAAAGTTCGCGTGATGGCAGCCGATTATGAAAATCAGCTCGACGAGTTTTCCCTCGATGCG
CCCATTGTCGCACATGAAAACTATCAATGGGCTAACTACGTTCGTGGCGTGGTGAAACATCTGCAACTGCGTAACAAC
AGCTTCGGCGGCGTGGACATGGTGATCAGCGGCAATGTGCCGACGGGTGCCGGGTTAAGTTCTTCCGCTTCACTGGAA
GTCGCGGTCGGAACCGTATTGCAGCAGCTTTATCATCTGCCGCTGGACGGCGCACAAATCGCGCTTAACGGTCAGGAA
GCAGAAAACCAGTTTGTAGGCTGTAACTGCGGGATCATGGATCAGCTAATTTCCGCGCTCGGCAAGAAAGATCATGCC
TTGCTGATCGATTGCCGCTCACTGGGGACCAAAGCAGTTTCCATGCCCAAAGGTGTGGCTGTCGTCATCATCAACAGT
AACTTCAAACGTACCCTGGTTGGCAGCGAATACAACACCCGTCGTGAACAGTGCGAAACCGGTGCGCGTTTCTTCCAG
CAGCCAGCCCTGCGTGATGTCACCATTGAAGAGTTCAACGCTGTTGCGCATGAACTGGACCCGATCGTGGCAAAACGC
GTGCGTCATATACTGACTGAAAACGCCCGCACCGTTGAAGCTGCCAGCGCGCTGGAGCAAGGCGACCTGAAACGTATG
GGCGAGTTGATGGCGGAGTCTCATGCCTCTATGCGCGATGATTTCGAAATCACCGTGCCGCAAATTGACACTCTGGTA
GAAATCGTCAAAGCTGTGATTGGCGACAAAGGTGGCGTACGCATGACCGGCGGCGGATTTGGCGGCTGTATCGTCGCG
CTGATCCCGGAAGAGCTGGTGCCTGCCGCACAGCAAGCTGTCGCTGAACAATATGAAGCAAAAACAGGTATTAAAGAG
ACTTTTTACGTTTGTAAACCATCACAAGGAGCAGGACAGTGCTGAACGAAACTCCCGCACTGCAGGATCgatcCATAT
GACGTCGACGCGTCTGCAGAAGCTTCTAGGTGAATTCAGGTGGAGTACGCGCCCGGGGAGCCCAAGGGCACGCCCTGG
CACCCGCACCGCGGGAGCTCCCGGGTACCAtggCGGTGAACAGTTGTTCTACTTTTGTTTGTTAGTCTTGATGCTTCA
CTGATAGATACAAGAGCCATAAGAACCTCAGATCCTTCCGTATTTAGCCAGTATGTTCTCTAGTGTGGTTCGTTGTTT
TTGCGTGAGCCATGAGAACGAACCATTGAGATCATGCTTACTTTGCATGTCACTCAAAAATTTTGCCTCAAAACTGGT
GAGCTGAATTTTTGCAGTTAAAGCATCGTGTAGTGTTTTTCTTAGTCCGTTACGTAGGTAGGAATCTGATGTAATGGT
TGTTGGTATTTTGTCACCATTCATTTTTATCTGGTTGTTCTCAAGTTCGGTTACGAGATCCATTTGTCTATCTAGTTC
AACTTGGAAAATCAACGTATCAGTCGGGCGGCCTCGCTTATCAACCACCAATTTCATATTGCTGTAAGTGTTTAAATC
TTTACTTATTGGTTTCAAAACCCATTGGTTAAGCCTTTTAAACTCATGGTAGTTATTTTCAAGCATTAACATGAACTT
AAATTCATCAAGGCTAATCTCTATATTTGCCTTGTGAGTTTTCTTTTGTGTTAGTTCTTTTAATAACCACTCATAAAT
CCTCATAGAGTATTTGTTTTCAAAAGACTTAACATGTTCCAGATTATATTTTATGAATTTTTTTAACTGGAAAAGATA
AGGCAATATCTCTTCACTAAAAACTAATTCTAATTTTTCGCTTGAGAACTTGGCATAGTTTGTCCACTGGAAAATCTC

```
AAAGCCTTTAACCAAAGGATTCCTGATTTCCACAGTTCTCGTCATCAGCTCTCTGGTTGCTTTAGCTAATACACCATA
AGCATTTTCCCTACTGATGTTCATCATCTGAGCGTATTGGTTATAAGTGAACGATACCGTCCGTTCTTTCCTTGTAGG
GTTTTCAATCGTGGGGTTGAGTAGTGCCACACAGCATAAAATTAGCTTGGTTTCATGCTCCGTTAAGTCATAGCGACT
AATCGCTAGTTCATTTGCTTTGAAAACAACTAATTCAGACATACATCTCAATTGGTCTAGGTGATTTTAATCACTATA
CCAATTGAGATGGGCTAGTCAATGATAATTACTAGTCCTTTTCCTTTGAGTTGTGGGTATCTGTAAATTCTGCTAGAC
CTTTGCTGGAAAACTTGTAAATTCTGCTAGACCCTCTGTAAATTCCGCTAGACCTTTGTGTGTTTTTTTTGTTTATAT
TCAAGTGGTTATAATTTATAGAATAAAGAAAGAATAAAAAAAGATAAAAAGAATAGATCCCAGCCCTGTGTATAACTC
ACTACTTTAGTCAGTTCCGCAGTATTACAAAAGGATGTCGCAAACGCTGTTTGCTCCTCTACAAAACAGACCTTAAAA
CCCTAAAGGCTTAAGTAGCACCCTCGCAAGCTCGGGCAAATCGCTGAATATTCCTTTTGTCTCCGACCATCAGGCACC
TGAGTCGCTGTCTTTTTCGTGACATTCAGTTCGCTGCGCTCACGGCTCTGGCAGTGAATGGGGGTAAATGGCACTACA
GGCGCCTTTTATGGATTCATGCAAGGAAACTACCCATAATACAAGAAAAGCCCGTCACGGGCTTCTCAGGGCGTTTTA
TGGCGGGTCTGCTATGTGGTGCTATCTGACTTTTTGCTGTTCAGCAGTTCCTGCCCTCTGATTTTCCAGTCTGACCAC
TTCGGATTATCCCGTGACAGGTCATTCAGACTGGCTAATGCACCCAGTAAGGCAGCGGTATCATCAACAGGCTTACCC
GTCTTACTGTCCCGGATCCGTCGACCTGCAGGGGGGGGGGGGCGCTGAGGTCTGCCTCGTGAAGAAGGTGTTGCTGAC
TCATACCAGGCCTGAATCGCCCCATCATCCAGCCAGAAAGTGAGGGAGCCACGGTTGATGAGAGCTTTGTTGTAGGTG
GACCAGTTGGTGATTTTGAACTTTTGCTTTGCCACGGAACGGTCTGCGTTGTCGGGAAGATGCGTGATCTGATCCTTC
AACTCAGCAAAAGTTCGATTTATTCAACAAAGCCGCCGTCCCGTCAAGTCAGCGTAATGCTCTGCCAGTGTTACAACC
AATTAACCAATTCTGATTAGAAAAACTCATCGAGCATCAAATGAAACTGCAATTTATTCATATCAGGATTATCAATAC
CATATTTTTGAAAAAGCCGTTTCTGTAATGAAGGAGAAAACTCACCGAGGCAGTTCCATAGGATGGCAAGATCCTGGT
ATCGGTCTGCGATTCCGACTCGTCCAACATCAATACAACCTATTAATTTCCCCTCGTCAAAAATAAGGTTATCAAGTG
AGAAATCACCATGAGTGACGACTGAATCCGGTGAGAATGGCAAAAGCTTATGCATTTCTTTCCAGACTTGTTCAACAG
GCCAGCCATTACGCTCGTCATCAAAATCACTCGCATCAACCAAACCGTTATTCATTCGTGATTGCGCCTGAGCGAGAC
GAAATACGCGATCGCTGTTAAAAGGACAATTACAAACAGGAATCGAATGCAACCGGCGCAGGAACACTGCCAGCGCAT
CAACAATATTTTCACCTGAATCAGGATATTCTTCTAATACCTGGAATGCTGTTTTCCCGGGGATCGCAGTGGTGAGTA
ACCATGCATCATCAGGAGTACGGATAAAATGCTTGATGGTCGGAAGAGGCATAAATTCCGTCAGCCAGTTTAGTCTGA
CCATCTCATCTGTAACATCATTGGCAACGCTACCTTTGCCATGTTTCAGAAACAACTCTGGCGCATCGGGCTTCCCAT
ACAATCGATAGATTGTCGCACCTGATTGCCCGACATTATCGCGAGCCCATTTATACCCATATAAATCAGCATCCATGT
TGGAATTTAATCGCGGCCTCGAGCAAGACGTTTCCCGTTGAATATGGCTCATAACACCCCTTGTATTACTGTTTATGT
AAGCAGACAGTTTTATTGTTCATGATGATATATTTTTATCTTGTGCAATGTAACATCAGAGATTTTGAGACACAACGT
GGCTTTCCCCCCCCCCCCTGCAG
```

pFM154 φC31 integrase attR x attL deletion substrate

pFM154 has identical sequence to pFM152 but with attP and attB sequences replaced with attR and attL respectively, in the same (direct repeat) orientation.

References

Proudfoot, C.M., McPherson, A.L., Kolb, A.F., and Stark, W.M. (2011) Zinc finger recombinases with adaptable DNA sequence specificity. *PLoS ONE 6*, e19537.

Olorunniji, F.J., McPherson, A.L., Rosser, S.J., Smith, M.C.M., Colloms, S.D., Stark, W.M. (2017) Control of serine integrase recombination directionality by fusion with the directionality factor. *Nucleic Acids Res. 45*, 8635-8645.
